# Target site selection and P1 engineering enable highly efficient circular RNA production via end-to-end self-targeting and splicing

**DOI:** 10.1093/nar/gkag869

**Published:** 2026-09-04

**Authors:** Kyung Hyun Lee, Seongcheol Kim, Juwon Cha, Seong-Wook Lee

**Affiliations:** R&D Center, Rznomics Inc., Seongnam 13468, Republic of Korea; R&D Center, Rznomics Inc., Seongnam 13468, Republic of Korea; R&D Center, Rznomics Inc., Seongnam 13468, Republic of Korea; Department of Bioconvergence Engineering, Research Institute of Advanced Omics, Dankook University, Yongin 16890, Republic of Korea; R&D Center, Rznomics Inc., Seongnam 13468, Republic of Korea; Department of Bioconvergence Engineering, Research Institute of Advanced Omics, Dankook University, Yongin 16890, Republic of Korea

## Abstract

Circular RNAs (circRNAs) are more stable than linear RNAs, enabling expanding applications in RNA vaccines and therapeutics. We previously developed an *in vitro* circRNA preparation method based on end-to-end self-targeting and splicing (STS) using the *Tetrahymena* group I intron, which generates circRNAs without extraneous sequences. However, self-circularization efficiency declines as gene of interest (GOI) length increases, limiting its application to longer GOIs. Here, we systematically optimized key determinants of STS efficiency, including target site selection and P1 construct engineering. Target site screening revealed that selection of optimal target sites within each GOI markedly improved self-circularization efficiency. Moreover, engineering of the P1 construct, including incorporation of a polyA10 sequence upstream of the internal guide sequence of the intron and an antisense sequence complementary to the target site and its upstream region at the 5′ side of polyA10, further enhanced efficiency. Notably, the optimized STS strategy achieved up to two-fold higher self-circularization efficiency than the conventional permuted intron–exon (PIE) method for long GOIs (∼8 K-nt). Collectively, these results establish an improved STS workflow for efficient circRNA production without extraneous sequences across a wide range of GOI lengths, outperforming the PIE method for long GOIs, and broadening biomedical applications.

## Graphical Abstract

**Graphical Abstract:**
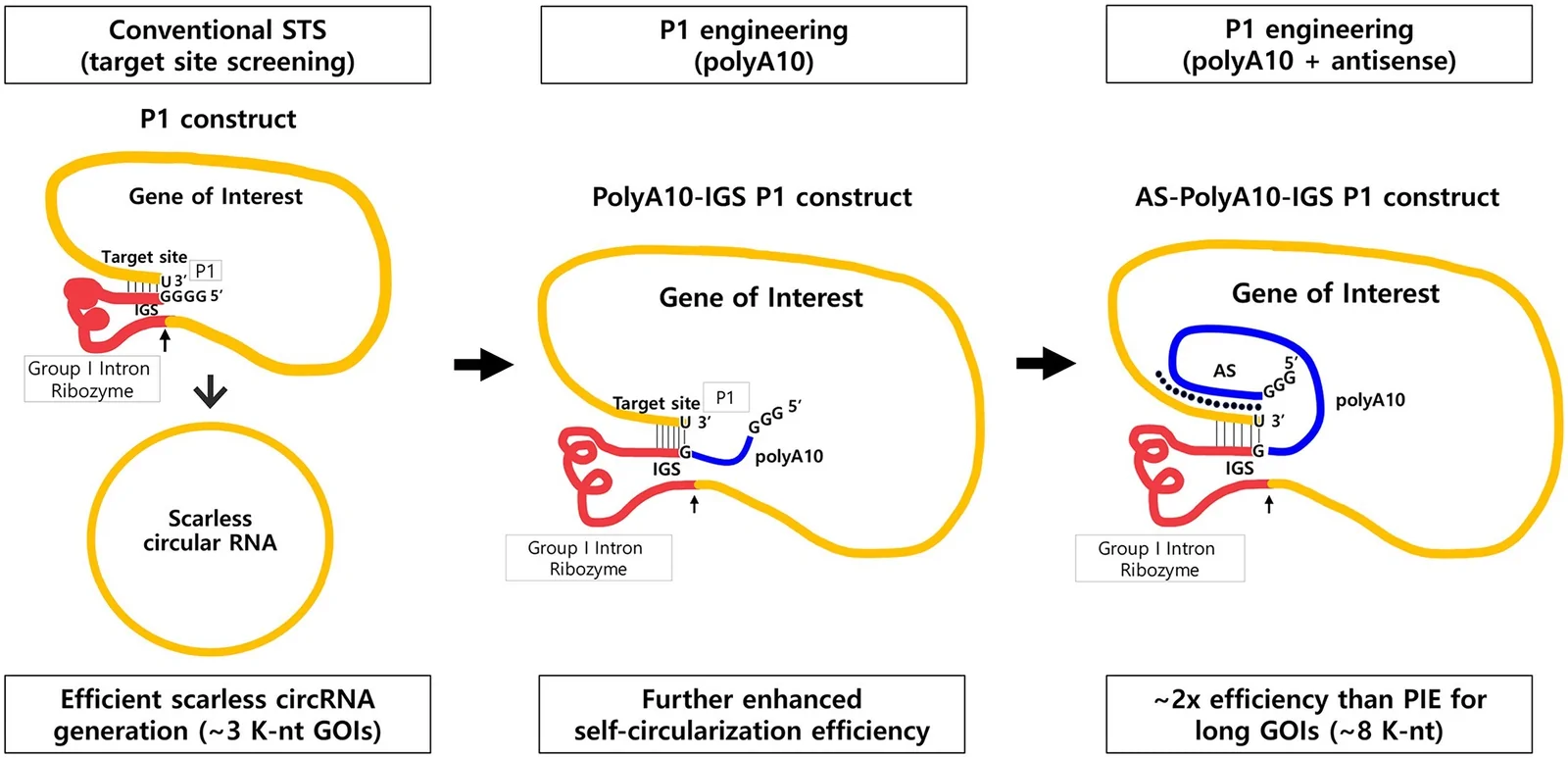

## Introduction

Circular RNAs (circRNAs) are covalently closed RNA molecules that differ from linear RNAs by the absence of free 5′ and 3′ ends [1]. Although circRNAs were initially regarded as random splicing byproducts, it is now evident that they are abundant and functionally important in many eukaryotic organisms [2–4]. Their closed structures confer resistance to exonuclease-mediated degradation, resulting in enhanced stability compared with linear transcripts. This increased stability makes circRNAs attractive for a wide range of applications, including gene regulation, diagnostics, and therapeutics [4,5]. Accordingly, circRNAs have recently been actively explored for biomedical applications, such as vaccines [6–9], RNA editing [10], and chimeric antigen receptor development [11], etc.

Despite their advantages over linear RNAs in various biomedical applications [5], conventional *in vitro* circRNA preparation methods have several limitations [5,12,13]. The most widely used practical approach is the permuted intron–exon (PIE) method, which strategically rearranges group I intron segments to induce intramolecular splicing and generate circRNAs [14,15]. However, the PIE method introduces E1/E2 fragments (i.e. intronic scar) that are required only during self-circularization reaction but remain as extraneous sequences in the final circRNA product [15]. In addition, spacer sequences that are also additional sequences to GOI are often required to improve the self-circularization efficiency of PIE method. This requirement arises because the 3′ PIE splice site is located near internal ribosome entry site (IRES), which may interfere with proper folding of the group I intron ribozyme [15]. Previous report suggested that circRNAs generated by the PIE method may provoke unwanted immune responses due to extraneous sequences (E1/E2 and spacers) that can form stable intramolecular RNA duplexes [16]. To circumvent these sequence constraints, Rausch *et al*. systematically characterized the E1/E2 sequence requirements of the PIE method and established design guidelines for selecting an optimal end-joining junction within the GOI by permuting the RNA sequence while retaining the PIE framework [17].

Alternatively, the self-targeting and splicing (STS) method uses the*Tetrahymena* group I intron ribozyme to ligate the 5′ and 3′ ends of the GOI during *in vitro* transcription (IVT) [18]. Since the STS method allows selection of the target site from within the GOI region and the group I intron is completely released out after the self-circularization reaction, it leaves no extraneous sequences, including spacers [19]. Consequently, *in vitro* synthesized circRNAs identical to natural circRNAs can be generated without additional sequences, fully maintaining their biological function in cells [20]. Recently, a novel self-splicing system based on the *Tetrahymena* group I intron, in which the ribozyme core is positioned at one end of the precursor and homology arm-like sequences are incorporated, thereby reconstituting a P9.0-like interaction derived from the native intron structure, also demonstrated the production of scarless circRNAs without spacer sequences [21]. Nevertheless, efficient self-circularization, particularly for long GOIs, remains a major challenge for practical *in vitro* circRNA production, and systematic strategies to improve STS-mediated self-circularization efficiency have not yet been established. Therefore, improving self-circularization efficiency is an important prerequisite for scalable and practical circRNA manufacturing.

Previous studies on the STS method have not systematically investigated how the selection of target sites within the GOI influences self-circularization efficiency [18,20]. In this study, we examine how different target site positions affect the self-circularization efficiency of various GOIs in the end-to-end STS reactions. Moreover, we developed new strategies to complement the laborious target site screening process by engineering the P1 self-circularization construct. These optimizations enable more efficient circRNA production for long GOIs of up to ∼8 K-nt, compared with the PIE method.

In summary, we systematically investigated the effects of target site selection and P1 construct engineering on self-circularization efficiency in the end-to-end STS reaction. Our findings provide practical guidance for efficient STS-based *in vitro* circRNA production across diverse biomedical applications.

## Materials and methods

### Preparation of self-circularization RNA constructs

DNAs encoding self-circularization RNA constructs [18] and PIE constructs [15] were synthesized using the gBlocks gene synthesis service (Integrated DNA Technologies). The synthesized DNA fragments were cloned into the pTOP vector using the TOPcloner TA-Blunt Kit (Enzynomics) or the In-Fusion HD cloning kit (Takara Bio) according to the manufacturers’ instructions. All constructs were verified by Sanger sequencing (Cosmogenetech). DNA templates for T7 IVT were generated by standard PCR using a thermal cycler and a Phusion Hot Start II High-Fidelity DNA polymerase (Thermo Fisher Scientific). The T7 G F primer (5′-ATAATACGACTCACTATAGGGG-3′) and a construct-specific R primer were used. Each R primer contained 20 to 40 nt of reverse complementary sequence corresponding to the selected target site located at the 3′ end of GOI. For example, the R primer sequence 5′-AATAAAATGAAACACGGACACCCAA-3′ was used to generate a T7 DNA template containing the AU11 target site from the coxsackievirus (CVB3) IRES. When the AU16 target sequence (5′-AUUUAU-3′) was additionally introduced into a specific region of GOI, the reverse complementary sequence of the AU16 target sequence was incorporated at the 5′-end of R primer [e.g. 5′-ATAAATTCGCCTTCAAATTTCACTTCCGCAC-3′ for the R primer for T7 DNA template containing the additional AU16 target sequence (5′-AUUUAU-3′) in the coding sequence (CDS) region of the GOI]. PCR products were purified using a LaboPass gel extraction kit (Cosmogenetech). T7 DNA templates for P1 construct engineering were also prepared using the primer sets described in Supplementary Table S1.

Various CDSs were used for self-circularization, including firefly luciferase (FLuc, CDS from TriLink BioTechnologies: Catalog No. L-7202 for the CVB3-FLuc P1 construct using the AU11 target site and CDS from Addgene #18 964 for the CVB3-FLuc P1 construct using the spacer target site, Renilla luciferase (RLuc) mutant M185V/Q235A [22], d2EGFP (destabilized EGFP, CDS from Clontech pd2EGFP-N1), gaussia luciferase (GLuc) [15], erythropoietin (EPO, CDS from TriLink BioTechnologies: Catalog No. L-7209), omicron spike protein (CDS from BioNTech mRNA vaccine BNT162b2), dCas9 (CDS from Addgene #47 316), and factor VIII (CDS from Addgene #41 036). CVB3 IRES was used to drive translation of the CDS.

### Circular RNA generation

CircRNAs were generated via self-circularization during IVT under the following conditions. IVT was carried out using PCR-generated T7 DNA templates and the HiScribe^TM^ T7 High Yield RNA synthesis kit (New England Biolabs) according to the manufacturer’s instructions. The resulting IVT products, following DNase I (Roche) treatment (1 unit/500 ng of DNA template), were purified using a Monarch RNA cleanup kit (New England Biolabs). For enrichment of circRNA, selected IVT samples were treated with RNase R (MCLAB) according to the manufacturer’s instructions with minor modification (120 μg of IVT sample/20 units of RNase R) for 30 min at 37°C. RNase R-treated samples were subsequently purified using a Monarch RNA cleanup kit. The PIE method was performed as previously described [15].

### Circular RNA analysis by gel electrophoresis

IVT samples were analyzed using denaturing polyacrylamide gel electrophoresis (PAGE) or E-gel^TM^ electrophoresis. CircRNA could be distinguished from linear RNA of the same size based on its slower migration.

For denaturing 4% polyacrylamide-7 M urea gel electrophoresis (denaturing PAGE, 20 × 20 cm gel size) using the PROTEAN II xi system (Bio-Rad), samples and size markers were mixed with 10 M urea-bromophenol blue loading dye in 1× Tris-Borate-EDTA buffer and heated at 75°C for 5 min prior to loading. Gel electrophoresis was performed at 50°C under 50 W constant power using a PowerPac HV power supply and temperature probe (Bio-Rad). After electrophoresis, gel was stained with SYBR Gold nucleic acid gel stain (Invitrogen) and analyzed using an ImageQuant800 imager (Cytiva). As size markers, a 100 bp Plus DNA ladder (Bioneer) and a RiboRuler Low Range RNA ladder (Thermo Fisher Scientific) were loaded alongside the samples. Under denaturing PAGE conditions, the DNA ladder migrated more slowly than the RNA ladder of the same size. Relative band intensity (%) of circRNA was calculated to compare the relative self-circularization efficiency [(Intensity of circRNA band/Intensity of lane) × 100].

For analysis using 1% E-gel^TM^ EX agarose gel (Invitrogen), gel electrophoresis was performed with the E-Gel^TM^ power snap electrophoresis system (Invitrogen) using the E-gel EX 1%-2% program according to the manufacturer’s instructions. An ssRNA ladder (New England Biolabs) was used as a size marker. The percentage of circRNA band was calculated using ImageJ software according to the formula [circRNA band intensity/(circRNA band intensity + intact precursor band intensity) × 100].

Denaturing PAGE was primarily used for relative comparison of self-circularization efficiency among relatively small constructs because of its high resolution, whereas E-gel electrophoresis was applied to large constructs that could not be readily resolved by denaturing PAGE.

### Analysis of circular RNA splicing junction sequences

RT-PCR was performed to verify the sequence of the splicing junction using specific primers, the sequences of which are provided in Supplementary Table S1. For reverse transcription, OneScript plus reverse transcriptase (Applied Biological Materials) was used according to the manufacturer’s instructions with 100 ng of RNA as input. The resulting complementary DNAs were subjected to 35 cycles of standard PCR using AccuPower Taq PCR premix (Bioneer). The amplified band of the expected size was excised from 2% agarose gel and purified using a LaboPass gel extraction kit (Cosmogenetech). Purified PCR products were cloned using the TOPcloner TA-Blunt kit (Enzynomics), followed by Sanger sequencing performed by Cosmogentech.

### Circular RNA analysis and purification by HPLC

In vitro transcribed circRNAs with or without RNase R digestion were analyzed or purified using ion-pair reversed-phase high-performance liquid chromatography (IP-RP HPLC). For analytical runs, equal amounts of IVT samples were injected into a 1260 Prime LC system (Agilent Technologies) equipped with a 250 × 4.6 mm reverse-phase LC column with a particle size of 5 μm and a pore size of 4000 Å (Agilent Technologies: PLRP-S 4000 Å). RNA was separated using a linear gradient using buffer A [100 mM hexylammonium acetate (HAA) in 10% acetonitrile (ACN); ADS Biotec] and buffer B (100 mM HAA in 50% ACN; ADS Biotec) at a flow rate of 0.7 ml/min. The column oven temperature was maintained at 60°C. RNA was detected by UV absorbance at 260 nm, and chromatograms were analyzed using OpenLab CDS software version 2.7 (Agilent Technologies). For circularization efficiency comparison in HPLC analysis, IVT samples were analyzed, and peak regions encompassing multiple peaks, including those of the intact precursor and circRNA, were defined using the Integration Optimizer function, while peaks corresponding to the cleaved ribozyme were excluded. The area of each peak was then automatically calculated. The percentage of each peak relative to the total measured peaks was also automatically determined by the software, and the percentage of the circRNA peak was used as the relative circularization efficiency for comparison. IP-RP HPLC served as an orthogonal analytical method for circRNA quantification based on the relative area of the circRNA peak. Because denaturing PAGE, E-gel electrophoresis, and IP-RP HPLC employ different separation principles and quantification approaches, self-circularization efficiencies should be interpreted only within each analytical method and should not be directly compared across methods.

For purification of circRNA for cell-based assay, RNase R-treated IVT samples were purified using a 1260 Infinity II bio-inert LC system (Agilent Technologies) equipped with a 250 × 10 mm reverse-phase LC column with a particle size of 5 μm and a pore size of 4000 Å (Agilent Technologies: PLRP-S 4000 Å). RNA was purified using the same linear gradient condition. The flow rate was set at 1.8 ml/min, and the column oven temperature was maintained at 60°C. RNA was monitored by UV absorbance at 260 nm. Fractions corresponding to circRNA were collected and recovered by the standard ethanol precipitation method. The recovered circRNAs were dissolved in nuclease-free water, and concentrations were determined using a NanoDrop microvolume UV-Vis spectrophotometer (Thermo Fisher Scientific).

### Cell-based luciferase assay

To evaluate circRNA expression following transfection, cell-based RLuc, FLuc, or superfolder (sGFP) assays were performed. The adenoviral E1-transformed human embryonic kidney cell line HEK293A (Invitrogen) was maintained in DMEM (Invitrogen) supplemented with 10% fetal bovine serum (Cytiva). For circRNA transfection, 2 × 10^4^ cells/well were seeded into 96-well white opaque plates (Thermo Fisher Scientific). Equimolar amounts (∼100 ng) of each CVB3-RLuc mutant M185V/Q235A [22] circRNA (a destabilized variant relative to the wild-type RLuc), CVB3-FLuc circRNA, or CVB3-sGFP circRNA were transfected using Lipofectamine MessengerMAX (Invitrogen) according to the manufacturer’s instructions. Translation of the reporter proteins was driven by the CVB3 IRES. RLuc or FLuc activities were measured 48 h after transfection using an RLuc assay system and an FLuc assay system (Promega), respectively. Luminescence was detected with a Synergy H1 microplate reader (BioTek). For sGFP, fluorescence intensity was measured 24 h after transfection using the same microplate reader. Each condition was tested in six replicates.

## Results

### Position-dependent effects of an identical target sequence on self-circularization efficiency

**Figure 1.**
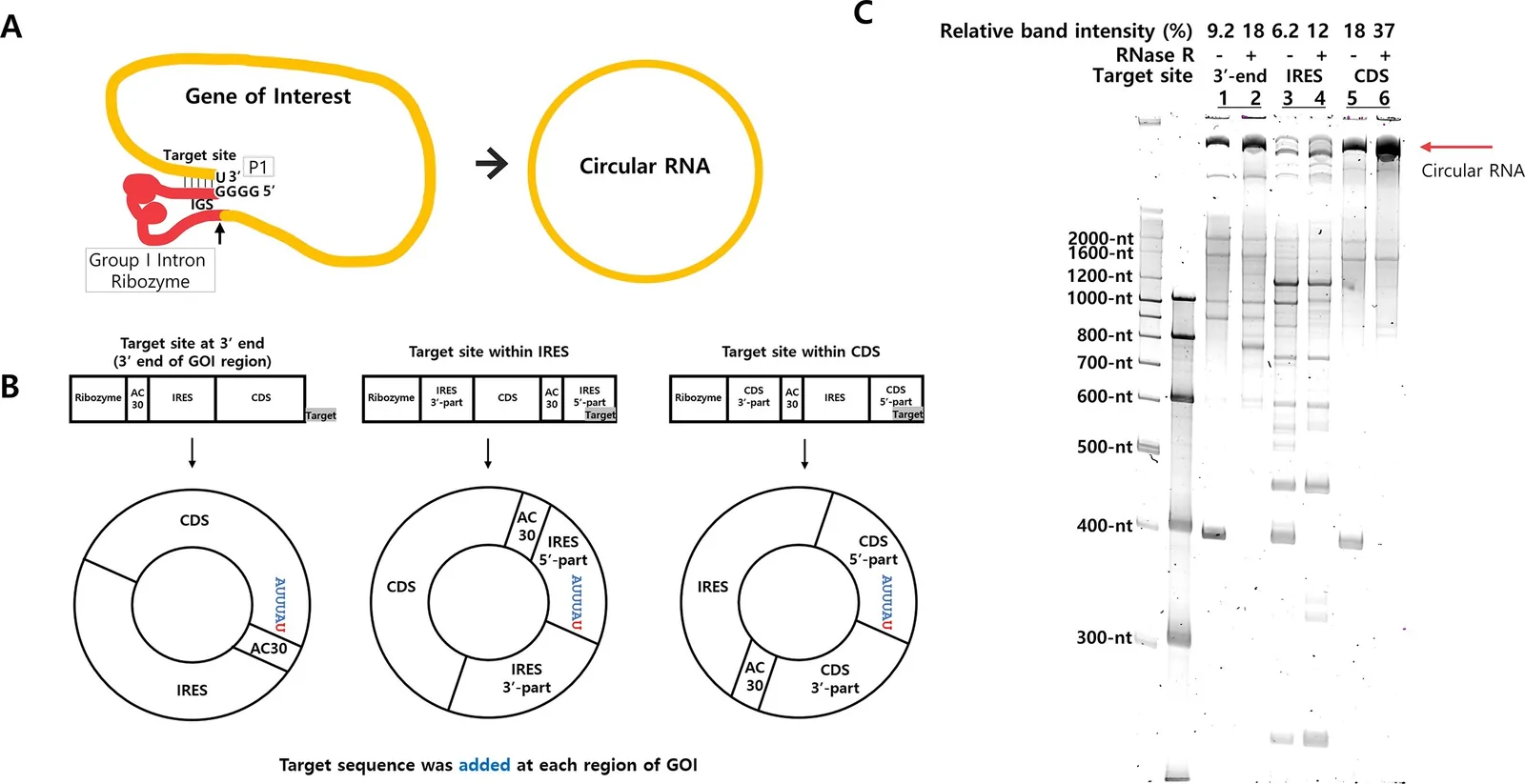
Self-circularization efficiencies of P1 constructs for CVB3-sGFP-AC30 GOI. (**A**) Schematic of the P1 construct for self-circularization via the end-to-end STS reaction. During IVT using T7 RNA polymerase, transcription initiates with the 5′-GGG sequence derived from the T7 promoter, resulting in a precursor RNA containing a 5′-terminal GGG sequence. In the P1 construct, the IGS pairs with the target site within the GOI to form the P1 helix, enabling self-circularization through a transesterification reaction. Any position within the GOI can be selected as a target site for self-circularization. The split GOI parts can be reconnected in the correct order following circRNA generation. CVB3-sGFP-AC30 constructs with the same target sequence positioned at various regions within the GOI (B, C). (**B**) Design of P1 constructs for self-circularization. The same target sequence (AU16: 5′-AUUUAU-3′) was introduced into various regions of the GOI, including at the 3′-end of the CDS, within the IRES, or within the CDS. AC30 refers to the AC30 spacer. (**C**) 4% denaturing PAGE analysis of IVT samples (lanes 1, 3, and 5) or RNase R-treated IVT samples (lanes 2, 4, and 6) to compare relative self-circularization efficiencies of P1 constructs containing the same target sequence at different positions within the GOI. Lanes 1 and 2, AU16 target site at the 3′-end of the CDS. Lanes 3 and 4, AU16 target site within the CVB3 IRES. Lanes 5 and 6, AU16 target site within the CDS (sGFP). Relative band intensity (%) of circRNA was calculated as the proportion of the circRNA band relative to the total band intensity within each lane and used to compare relative self-circularization efficiency.

Previous studies on self-circularization using the STS method have demonstrated that a target site, which determines the splicing junction through interaction with the internal guide sequence (IGS), can be selected from various regions of GOI [18,20]. However, the effect of target site position from within the GOI on self-circularization efficiency has not been systematically investigated. To address this question, individual P1 constructs were designed for self-circularization (Fig. 1A). In our previous study [18], we systematically evaluated 32 AU-rich target sequences (AU1 to AU32) containing the 5′-WWWWWU-3′ motif (where W represents A or U), which generally exhibited higher self-circularization efficiencies than the non-AU-rich target sequences tested. The same AU-rich target sequence (AU16: 5′-AUUUAU-3′), which was previously shown to support efficient self-circularization [18], was introduced into various regions of GOI, including at the 3′-end of the CDS, within the IRES, or within the CDS itself (Fig. 1B). Their self-circularization efficiencies were then compared. The coxsackievirus B3 (CVB3) IRES, which is widely used in circRNA research due to its robust protein expression in various cell types [15,23,24], and superfolder GFP (sGFP) as the CDS were employed in this study. The target sites employed for self-circularization are shown in Supplementary Fig. S1A and B. An AC30 spacer (Supplementary Table S1) was inserted at the junction between the CDS terminus and the 5′ end of the IRES (CVB3-sGFP-AC30 circRNA).

Self-circularization efficiency of each P1 construct using the same AU16 target sequence at different positions within the GOI was analyzed by 4% denaturing PAGE following IVT (Fig. 1C). Self-circularization efficiency was highest when the AU16 target site was introduced within the CDS (18% in relative band intensity), whereas positioning the target site within the IRES resulted in the lowest efficiency (6.2% in relative band intensity). These results indicate that self-circularization efficiency varies markedly depending on the position of the target site within the GOI, even when the same target sequence is used.

To confirm the precise STS-mediated circRNA generation, sequencing analysis was performed after RT-PCR using circRNA-specific primers (Supplementary Table S1), as previously described [18]. This analysis confirmed the formation of the correct junction in the generated circRNAs (Supplementary Fig. S2A).

### Spacer flexibility influences position-dependent effects of target sites on self-circularization

**Figure 2.**
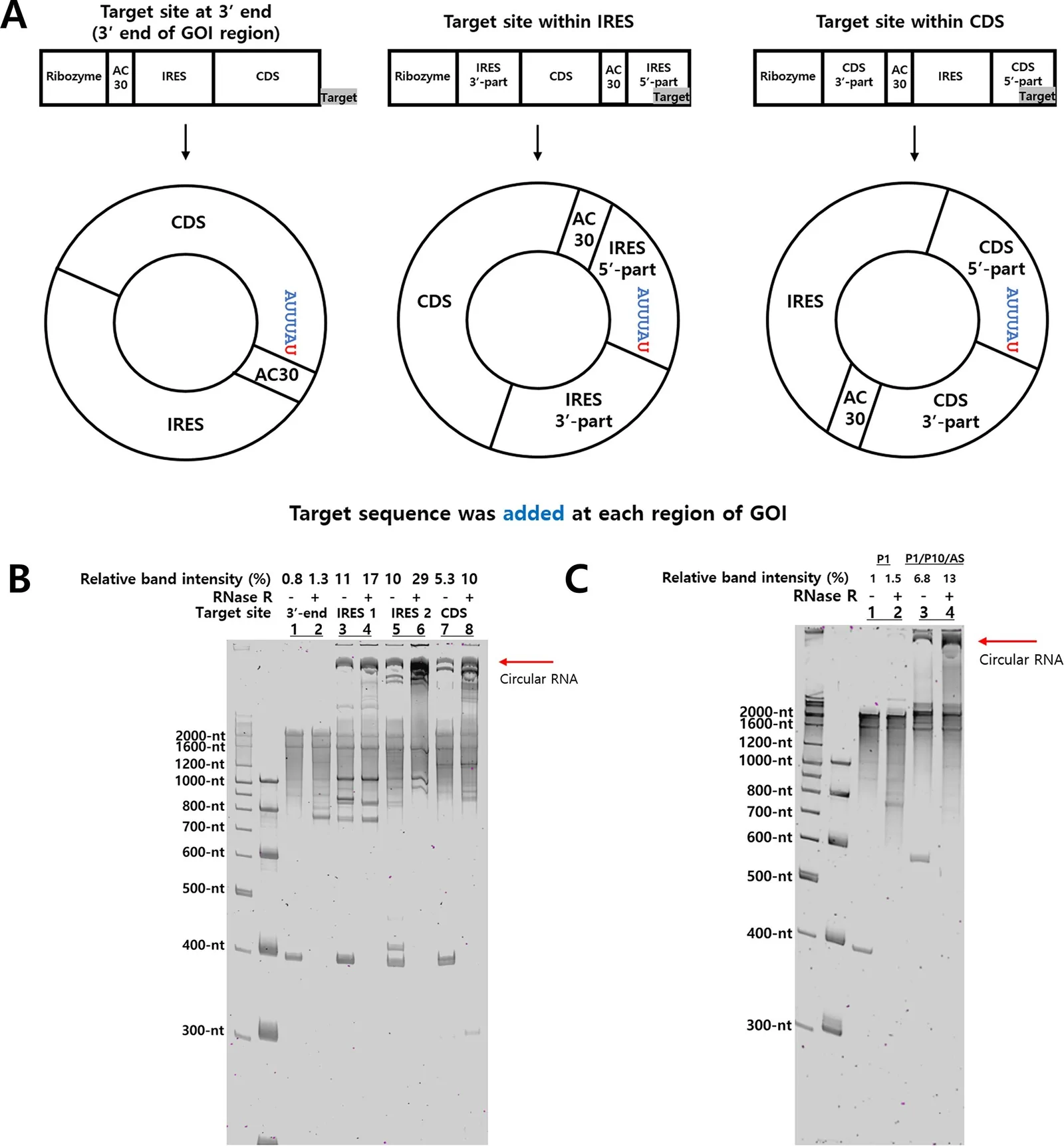
CVB3-sGFP-AC70 constructs with the different target sequences at various regions within the GOI were analyzed (A–C). (**A**) Design of P1 constructs using different target sequences. Target sequences were either introduced at the 3′-end of the CDS (AU16: 5′-AUUUAU-3′) or selected from specific regions of the GOI (2 sites from within the IRES or 1 site from within the CDS). The IRES 1 and 2 target site sequences were AU28 (5′-AUAUAU-3′) and 5′-GUAGAU-3′, respectively. The CDS target site was the AU32 target sequence (5′-AAAUUU-3′). For constructs with target sites located within the IRES or CDS, the target sequences correspond to the native GOI sequence. AC70 refers to the AC70 spacer. (**B**) 4% denaturing PAGE analysis of IVT (lanes 1, 3, 5, and 7) or RNase R-treated IVT samples (lanes 2, 4, 6, and 8) comparing relative self-circularization efficiencies of P1 constructs using different target sequences at various positions within the GOI. Lanes 1 and 2, AU16 target site at the 3′-end of the CDS. Lanes 3 and 4, IRES 1 target site (AU28) within the CVB3 IRES. Lanes 5 and 6, IRES 2 target site (5′-GUAGAU-3′) within the CVB3 IRES. Lanes 7 and 8, AU32 target site within the CDS (sGFP). (**C**) 4% denaturing PAGE analysis of IVT or RNase R-treated IVT samples comparing the P1 construct and the P1/P10/AS construct for the construct using the AU16 target site (5′-AUUUAU-3′) at the 3′-end of the CDS. Relative band intensity (%) of circRNA was calculated as the proportion of the circRNA band relative to the total band intensity within each lane and used to compare relative self-circularization efficiency.

Next, we examined whether different target sequences selected directly from within the GOI, with the exception of the target site at the 3′-end of the CDS, which was introduced externally, exhibit similar positional effects on self-circularization efficiency (Fig. 2A). In this experiment, the AC30 spacer used in Fig. 1A (CVB3-sGFP-AC30) was replaced with the AC70 spacer (Supplementary Table S1, CVB3-sGFP-AC70) to assess the effect of spacer length and sequence on self-circularization efficiency of the P1 construct using the AU16 target sequence (5′-AUUUAU-3′) at the 3′-end of the CDS (differing only in the spacer, AC30 or AC70). In addition, various target sites were selected directly from within the GOI (Fig. 2A). Accordingly, for P1 constructs with target sites positioned within the IRES or the CDS, the target site sequences were derived from the native GOI rather than introduced as additional sequences (Supplementary Fig. S1A and B).

Interestingly, while the P1 construct using the AU16 target site at the 3′-end of the CDS of the CVB3-sGFP-AC30 construct showed 9.2% of self-circularization efficiency in denaturing PAGE analysis (lane 1 of Fig. 1C), the corresponding construct with the AC70 spacer (CVB3-sGFP-AC70) showed only 0.8% efficiency of circRNA (lane 1 of Fig. 2B). These results suggest that spacer length and the resulting structural flexibility strongly influence self-circularization efficiency. Furthermore, the P1/P10/AS [antisense sequence (AS)] construct [18] (Supplementary Fig. S3), which has been shown to promote interaction between the IGS and the target site through additional complementary base pairing between the AS and the ABS (antisense binding sequence) and to enhance splicing reaction specificity and efficacy through a P10 helix incorporation [25], enabled self-circularization of 6.8% efficiency in denaturing PAGE analysis, when based on the CVB3-sGFP-AC70 backbone (Fig. 2C). In contrast, the corresponding P1 construct showed no significant self-circularization (1% efficiency, Fig. 2C). Based on observations from the CVB3-sGFP-AC70 GOI using P1 and P1/P10/AS construct designs, increased flexibility introduced by a longer spacer may spatially separate the IGS from the target site within the global RNA structure, thereby reducing efficient P1 helix formation (P1 construct). In this context, incorporation of the AS sequence may restore effective interaction between the IGS and the target site by bridging this separation through additional complementary base pairing (P1/P10/AS construct).

Consistent with earlier observations (Fig. 1B and C), different target sites positioned at distinct locations within the GOI exhibited varying self-circularization efficiencies (Fig. 2A and B). These findings further confirm that appropriate target site selection is a critical determinant of efficient STS-mediated circRNA preparation. Precise STS reaction for circRNA generation for each construct was also confirmed by sequencing analysis of the junction region (Supplementary Fig. S2B and C).

### AU11 Target site within the CVB3 IRES enables efficient self-circularization across diverse GOIs regardless of spacer length

We observed that the selection of an optimal target site within the GOI is critical for efficient self-circularization. Therefore, we sought to identify an optimal target site within the CVB3 IRES, which has been commonly used in circRNA studies because it enables efficient protein expression in various cell lines [15,23,24]. We hypothesized that use of an optimal target site within the CVB3 IRES could support efficient self-circularization regardless of the downstream CDS. In this set of experiments, RNA constructs containing the AC40 spacer were used (Supplementary Table S1).

**Figure 3.**
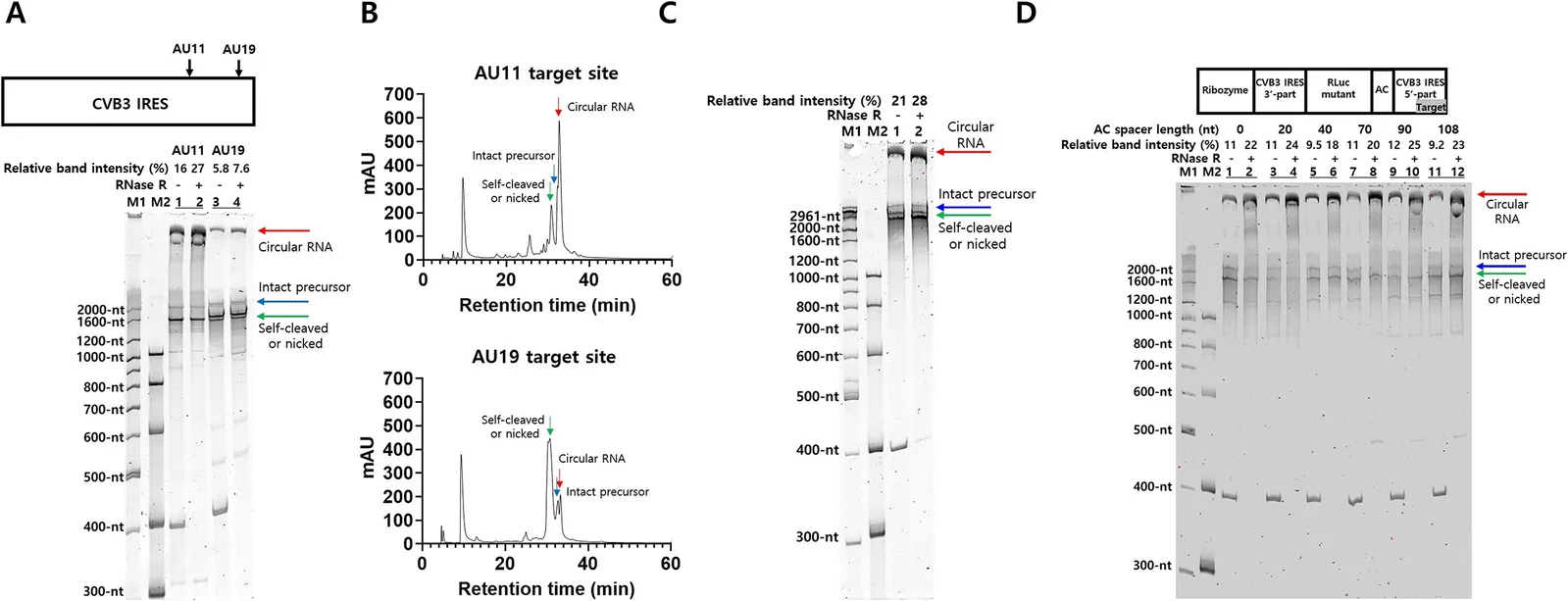
Self-circularization efficiencies of P1 constructs using AU11 or AU19 target site within the CVB3 IRES. (**A**) 4% denaturing PAGE analysis of IVT samples (lanes 1 and 3) or RNase R-treated IVT samples (lanes 2 and 4) comparing relative self-circularization efficiencies of P1 constructs (CVB3-sGFP-AC40) using the AU11 target site (lanes 1 and 2) or the AU19 target site (lanes 3 and 4). Relative band intensity (%) of circRNA was calculated as the proportion of the circRNA band relative to the total band intensity within each lane and used to compare relative self-circularization efficiency. (**B**) IP-RP HPLC analysis of IVT samples for each P1 construct. HPLC peaks corresponding to self-cleaved or nicked circRNA, intact precursor RNA, and circRNA are indicated by arrows (from left to right). (**C**) 4% denaturing PAGE analysis of IVT sample (lane 1) or the RNase R-treated IVT sample (lane 2) of the P1 construct for CVB3-FLuc circRNA using the AU11 target sequence within the CVB3 IRES and AC40 spacer. (**D**) 4% denaturing PAGE analysis of IVT samples (lanes 1, 3, 5, 7, 9, and 11) or RNase R-treated IVT samples (lanes 2, 4, 6, 8, 10, and 12) of P1 constructs for CVB3-RLuc mutant circRNAs using the AU11 target sequence within the CVB3 IRES and spacer sequences of varying lengths. AC refers to the AC spacer.

Additional AU-rich target sequences (AU11: 5′-UUUAUU-3′ or AU19: 5′-UAUUAU-3′) within the CVB3 IRES (Supplementary Fig. S1A) were tested in the P1 construct (CVB3-sGFP-AC40). AU11 target site showed substantially higher self-circularization efficiency (16% relative band intensity) compared with the AU19 target site (5.8%, Fig. 3A) and other IRES-derived target sites tested in Fig. 2. Although denaturing PAGE analysis provided relative quantification, IP-RP HPLC was performed for more accurate comparison, as peak areas corresponding to the intact precursor and circRNA can be quantitatively analyzed (Fig. 3B and Supplementary Fig. S4A and B), as previously described [18]. Consistently, the AU11 target site yielded a higher proportion of circRNA (57%) compared with the AU19 target site (13%), indicating that target site selection within the GOI strongly influences self-circularization efficiency even when both target sites are derived from within the same IRES. Precise STS-mediated circRNA generation of each construct was confirmed by sequencing analysis of the junction (Supplementary Fig. S2D).

We next evaluated whether the AU11 target site could support efficient self-circularization across diverse GOIs containing CVB3 IRES, with lengths up to approximately 3.5 K-nt. Efficient self-circularization was observed across the tested constructs. Representative examples with various CDSs are presented here, including FLuc, RLuc mutant M185V/Q235A [22], d2EGFP, GLuc [15], and EPO. Using AU11 target site, varying self-circularization efficiencies were observed across different GOIs, including the CVB3-sGFP construct (Fig 3A: 16% relative band intensity, Supplementary Fig. S4A: 57% circRNA peak area by HPLC), CVB3-FLuc (Fig. 3C and Supplementary Fig. S4C, 31% circRNA peak area), CVB3-RLuc mutant circRNA constructs (Fig. 3D, ∼10% relative band intensity across various spacer lengths), CVB3-GLuc (Supplementary Fig. S4D, 45% circRNA peak area), and CVB3-EPO (Supplementary Fig. S4E, 30% circRNA peak area), as confirmed by either denaturing PAGE or HPLC analysis.

We further examined whether spacer length influences self-circularization efficiency when AU11 target site is used, as spacer-dependent effects were observed in Figs 1 and 2. Spacer length markedly affected self-circularization efficiency when the AU16 target site was used for CVB3-sGFP constructs (lane 1 of Fig. 1C for AC30 and lane 1 of Fig. 2B for AC70), probably due to differences in global RNA conformation that influence the spatial proximity and interaction between the IGS and the target site. In sharp contrast, when AU11 target site within the CVB3 IRES was used for CVB3-RLuc mutant circRNA preparation, spacer sequences of varying lengths (AC20 to AC108) did not significantly affect self-circularization efficiency, suggesting that increased spacer length and the resulting structural flexibility do not substantially disrupt the interaction between the AU11 target site and the IGS, even up to 108 nt (Fig. 3D). RLuc mutant protein expression levels from circRNAs containing spacers of different lengths were comparable (Supplementary Fig. S5). As expected, two P1 constructs containing the same AC108 spacer but utilizing different target sites (AC108 for the AU11 target site or AC108* for spacer target site, Supplementary Table S1) produced similar levels of RLuc expression, as the resulting circRNAs contained identical GOI sequences regardless of target site selection (Supplementary Fig. S5). In the PIE method, spacer sequences are required to increase self-circularization efficiency by reducing structural interference between the IRES and ribozyme elements [15]. In contrast, the STS method does not require spacer sequences and avoids leaving intronic scars, provided that a structurally compatible target site can be selected [19]. Self-circularization efficiency was evaluated using both the STS and PIE methods with a spacer-free CVB3-FLuc GOI (Supplementary Fig. S6). Although the GOI was relatively short (2397-nt), the PIE method showed significantly lower self-circularization efficiency than the STS method for the same GOI, with relative band intensities of 9.9% and 14%, respectively. Nevertheless, AC spacers or untranslated region can be intentionally incorporated to enhance translational efficiency through interactions with RNA-binding proteins such as polyA-binding protein [23]. Precise STS-mediated circRNA generation was confirmed for all constructs by sequencing analysis of the junction, with a representative result shown in Supplementary Fig. S2E.

### P1 Construct engineering enhances self-circularization efficiency and complements target site screening

In general, longer GOI tend to exhibit reduced self-circularization efficiency compared with shorter GOIs [26]. Although target site screening can improve self-circularization efficiency for longer GOI, the process is labor-intensive and time-consuming, as multiple P1 constructs using various target sites from within the GOI must be designed, prepared, and evaluated individually. To improve self-circularization efficiency prior to extensive target site screening, or to further enhance efficiency in combination with target site screening, we developed a construct engineering strategy to optimize the group I intron ribozyme components within the P1 construct. This modification was explored to evaluate whether introducing defined sequence elements upstream of the IGS could modulate the local structure around the IGS via the extended sequence, thereby reducing nonspecific targeting or promoting efficient interaction with the target site. Engineering of the sequence upstream of the 5′ IGS was performed by PCR using specific primers (Supplementary Table S1), followed by IVT to evaluate the engineered P1 constructs.

For example, although AU11 target site in the CVB3 IRES was applied to longer GOIs, such as CVB3-Omicron spike protein (4569-nt) and CVB3-dCas9 (4851-nt), self-circularization efficiency was low for CVB3-Omicron spike protein (14% circRNA band intensity in E-gel analysis and 4.5% of circRNA peak area by HPLC, Supplementary Figs S7 and S8A). In contrast, the efficiency for CVB3-dCas9 was approximately three-fold higher than that of the CVB3-Omicron spike protein construct (37% in E-gel and 14% circRNA peak area by HPLC, Supplementary Figs S7 and S8B). These results indicate that self-circularization efficiency is not solely determined by GOI length, as constructs of comparable lengths, such as CVB3-Omicron spike and CVB3-dCas9, exhibited markedly different efficiencies, and is instead influenced by sequence context and overall RNA conformation. In particular, unfavorable conformation in certain GOIs, such as the CVB3-Omicron spike protein, may reduce effective interaction between the IGS and the target site. 

Because heating at 60°C is applied to the HPLC column to improve resolution during analysis, nick formation may occur, particularly in long GOIs. Therefore, self-circularization efficiencies should be interpreted comparatively across analytical methods (gel or HPLC), taking this factor into consideration.

**Figure 4.**
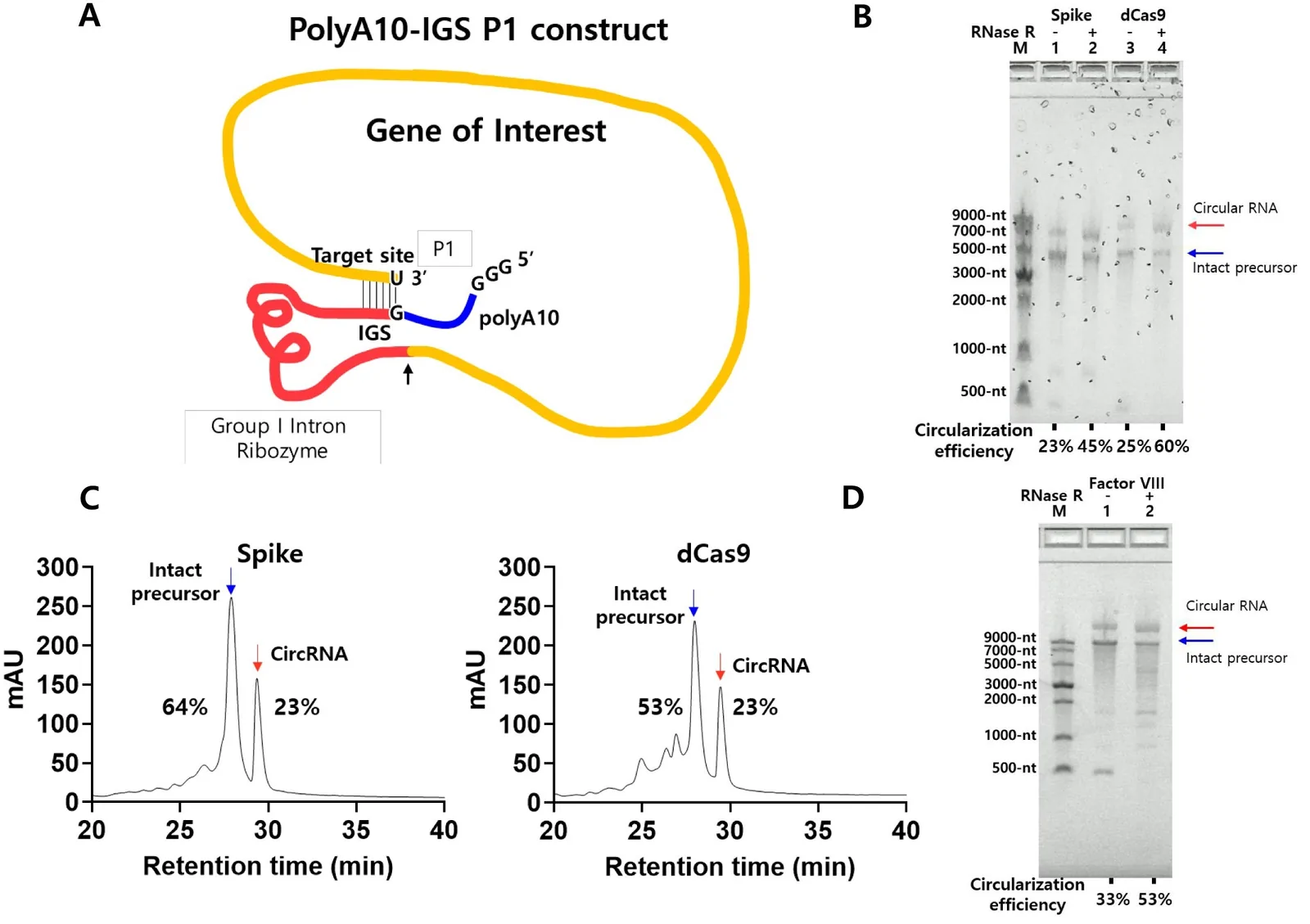
PolyA10-IGS P1 construct engineering strategy to improve self-circularization efficiency of the STS reaction. (**A**) Schematic of the PolyA10-IGS P1 construct containing a polyA10 sequence linked upstream of the IGS at its 5′ region. (**B**) 1% E-gel EX agarose gel analysis of IVT samples (lanes 1 and 3) or RNase R-treated IVT samples (lanes 2 and 4) from polyA10-IGS P1 constructs generating CVB3-Omicron spike and CVB3-dCas9 circRNAs, respectively. The percentage of the circRNA band was calculated as the relative proportion of circRNA to the sum of circRNA and intact precursor bands and was used as the relative circularization efficiency. (**C**) IP-RP HPLC analysis of IVT samples from each polyA10-IGS P1 construct generating CVB3-Omicron spike and CVB3-dCas9 circRNAs. HPLC peaks corresponding to intact precursor RNA and circRNA are indicated by arrows (from left to right). The percentage of peak area is shown next to each peak. The percentage of each peak relative to the total measured peaks was determined, and the percentage of the circRNA peak was used as the relative circularization efficiency for comparison. (**D**) 1% E-gel EX agarose gel analysis of IVT sample (lane 1) or RNase R-treated IVT sample (lane 2) from the polyA10-IGS P1 construct generating CVB3-Factor VIII circRNAP.

However, when a polyA10 sequence was introduced upstream of the IGS at its 5′ side as a construct engineering strategy (polyA10-IGS P1 construct, Fig. 4A), self-circularization efficiency increased for the CVB3-Omicron spike protein (23% in E-gel and 23% circRNA peak area by HPLC, Fig. 4B and C and Supplementary Fig. S9A) using the AU11 target site, compared with the conventional STS method (14% circRNA band intensity in E-gel analysis and 4.5% of circRNA peak area by HPLC, Supplementary Figs S7 and S8A). For CVB3-dCas9, self-circularization efficiency following the polyA10 modification was comparable to that of the original P1 construct (37% in E-gel and 14% circRNA peak area by HPLC, Supplementary Figs S7 and S8B) within experimental variation, showing 25% in E-gel and 23% circRNA peak area by HPLC. (Fig. 4B and C and Supplementary Fig. S9B). Precise STS-mediated circRNA generation for both constructs was confirmed by sequencing analysis of the junction (Supplementary Fig. S2F).

Moreover, the polyA10-IGS P1 construct design strategy was applicable to various GOIs and further improved self-circularization efficiency for both newly tested target sites and suboptimal target sites identified earlier in this study (Supplementary Fig. S10). For example, self-circularization efficiency for a suboptimal target site (spacer target site) in the CVB3-FLuc P1 construct (9.7% of circRNA peak area by HPLC) increased upon application of the polyA10-IGS P1 construct (52% of circRNA peak area). Similarly, for the CVB3-sGFP P1 construct using AU19 target site, the efficiency increased from 14% (Supplementary Fig. S4B) to 47% circRNA peak area in HPLC analysis (Supplementary Fig. S10). Using the polyA10-IGS strategy, CVB3-sGFP-AC70 circRNAs that had been inefficiently generated from different target sites in Fig. 2 were efficiently produced and purified for expression analysis in HEK293A cells. The resulting circRNAs had identical sequences regardless of the target site used for the STS reaction, except for the construct using the 3′-end target site, in which the AU16 target site (5′-AUUUAU-3′) was additionally introduced at the 3′-end (Fig. 2A). Nevertheless, all constructs showed comparable sGFP expression levels (Supplementary Fig. S11).

PolyA-IGS P1 constructs containing polyA sequences ranging from 5 to 30 nt did not significantly affect self-circularization efficiency (Supplementary Fig. S12), and polyA10 was therefore selected as the standard length. When polyA, U, G, and C sequences were tested, polyA yielded the highest efficiency, whereas the efficiencies of the other homopolymers varied depending on the GOI (Supplementary Fig. S13). The polyA10-IGS construct strategy was also applied to a 7800-nt CVB3-Factor VIII GOI without a spacer sequence (AC0). The factor VIII CDS contains a T-to-A substitution at nucleotide position 5157 to eliminate an internal AU11 target sequence, ensuring that the AU11 target sequence is present only in the CVB3 IRES. This construct showed improved self-circularization efficiency (33% in E-gel, Fig. 4D) compared with conventional STS IVT reaction without polyA10 (8% in E-gel, Supplementary Fig. S14). For long GOIs of ≥ 8 K-nt, IP-RP HPLC showed limited resolution for peak analysis and potential nick formation during heating-based analysis, and therefore self-circularization efficiency was assessed using E-gel only. Correct junction formation of circRNA generated using the polyA10-IGS construct was confirmed by sequencing analysis (Supplementary Fig. S2G).

To further characterize the sequence-dependent effects of the polyN region on STS-mediated self-circularization, additional 10-nt sequences with different base-pairing and self-complementarity potentials were tested while retaining the AU11 target site (5′-UUUAUU-3′) and its corresponding IGS sequence (5′-GAUAAA-3′) (Supplementary Fig. S15). Overall, polyN sequences with greater potential to form local base-paired or self-complementary structures such as poly(AU)5, poly(GC)5 and 5′-AAUUGGCCAU-3′, showed lower self-circularization efficiencies than polyA10 (4%-8% versus 23%, respectively, for both the CVB3-Omicron spike and CVB3-dCas9 constructs of Fig. 4C). In contrast, poly(AG)5, which has relatively low predicted self-complementarity and limited predicted complementarity to the upstream GGG sequence and the IGS, showed comparatively high and consistent self-circularization efficiencies in both CVB3-Omicron spike and CVB3-dCas9 constructs. However, this relationship was not absolute, as the highly self-complementary 5′-GCUAGCUAGC-3′ sequence retained self-circularization efficiency of 8%-12%, which was higher than those of several other polyN sequences with predicted complementary interactions (4%-8%).

### Incorporation of an antisense sequence into the P1 construct further enhances self-circularization efficiency

**Figure 5.**
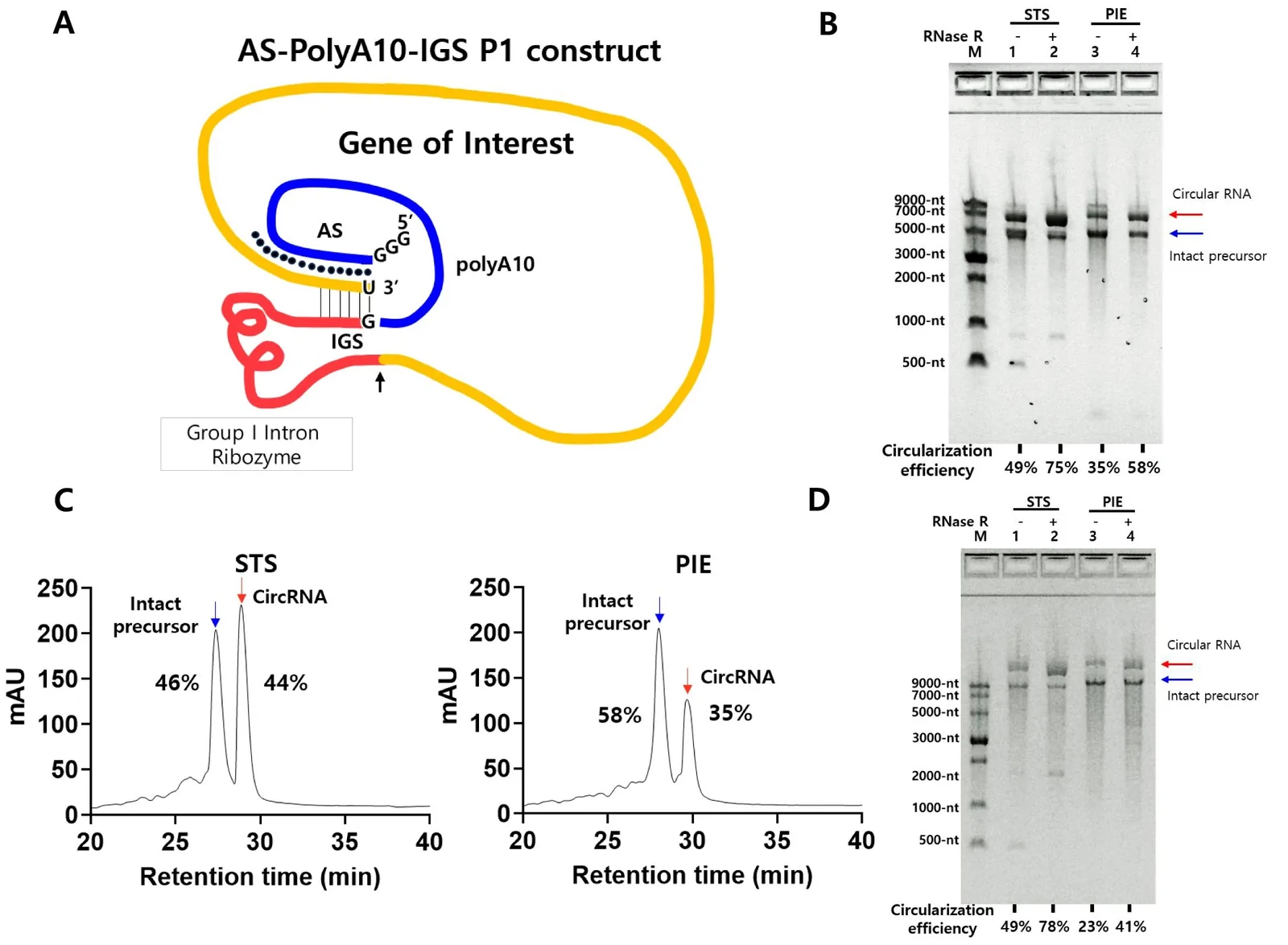
AS-polyA10-IGS P1 construct for further improvement of self-circularization efficiency. (**A**) Schematic of AS-polyA10-IGS P1 construct containing an AS-polyA10 sequence linked upstream of the IGS at its 5′ region. Dots indicate complementary base pairing of the AS with the target site and its surrounding sequences. (**B**) 1% E-gel EX agarose gel analysis of IVT samples (lanes 1 and 3) or RNase R-treated IVT samples (lanes 2 and 4) from the AS-polyA10-IGS P1 (STS) construct and the PIE construct generating CVB3-Omicron spike circRNA, respectively. The percentage of the circRNA band was calculated as the relative proportion of circRNA to the sum of circRNA and intact precursor bands and was used as the relative circularization efficiency. (**C**) IP-RP HPLC analysis of IVT samples from the AS-polyA10-IGS P1 (STS) construct and the PIE construct generating CVB3-Omicron spike circRNAs. HPLC peaks corresponding to intact precursor RNA and circRNA are indicated by arrows (from left to right). The percentage of peak is shown next to each peak. The percentage of each peak relative to the total measured peaks was determined, and the percentage of the circRNA peak was used as the relative circularization efficiency for comparison. (**D**) 1% E-gel EX agarose gel analysis of IVT sample (lanes 1 and 3) or RNase R-treated IVT sample (lanes 2 and 4) from the AS-polyA10-IGS P1 (STS) construct without a spacer (AC0) or the PIE construct with an intronic scar and a spacer (AC108) generating CVB3-Factor VIII circRNA.

As the polyA10-IGS P1 construct strategy increased self-circularization efficiency, we examined whether additional engineering could further enhance the efficiency. An AS was introduced at the 5′ side of the polyA10 sequence in the polyA10-IGS P1 construct to bind either the target site or the region immediately upstream of the target site at its 5′ side, depending on the design (AS-polyA10-IGS P1 construct; Fig. 5A and Supplementary Table S1), to evaluate whether additional complementary base paring could enhance the interaction between the IGS and the target site, thereby improving self-circularization efficiency. For example, AS6 binds directly to the AU11 target site (identical to the corresponding IGS sequence). AS8 binds to 8 nucleotides immediately upstream of the AU11 target site at its 5′ side without overlapping the target site. AS14 binds to the AU11 target site together with the adjacent 8 nucleotides upstream of the target site (Supplementary Fig. S16). Among these designs, the AS14-polyA10-IGS construct exhibited the highest self-circularization efficiency for the CVB3-Omicron spike GOI using the AU11 target site and is referred to as the AS-polyA10-IGS P1 construct unless otherwise indicated.

When the AS-polyA10-IGS P1 construct strategy was applied to the CVB3-Omicron spike GOI, an additional increase in self-circularization efficiency was observed (49% circRNA band intensity in E-gel analysis and 44% circRNA peak area by HPLC; Fig. 5B and C and Supplementary Fig. S17A), compared with the polyA10-IGS P1 construct (23% circRNA band intensity in E-gel analysis and 23% circRNA peak area by HPLC, Fig. 4B and C). This efficiency exceeded that achieved with the PIE method (35% in E-gel and 32% circRNA peak area by HPLC, Fig. 5B and C, Supplementary Fig. S17B). Moreover, when the AS-polyA10-IGS P1 construct for the CVB3-Factor VIII GOI (7800-nt) without a spacer sequence (AC0) was compared with the PIE method for the same GOI, which contains an intronic scar and an optimized spacer sequence (AC108) [15], self-circularization efficiency was approximately two-fold higher (49% circRNA band intensity in E-gel, Fig. 5D) than that of the PIE method (23% circRNA band intensity in E-gel, Fig. 5D). By combining optimized target site selection with P1 construct engineering, higher self-circularization efficiency was achieved for long GOIs up to two-fold (∼8 K-nt GOI) than with the conventional PIE method (Fig. 5C and D). The gel images and HPLC chromatograms shown in the figures are representative results, while the quantitative self-circularization efficiencies for long GOIs obtained from three independent IVT reactions using the P1, polyA10-IGS P1, AS-polyA10-IGS P1, and PIE strategies are summarized in Table 1 as mean ± SD.

**Table 1.** Comparison of relative self-circularization efficiencies of long GOIs generated by different circularization strategies

GOI	Analysis	STS Original P1	STS PolyA10-IGS	STS AS-PolyA10-IGS	PIE
CVB3-Omicron (4569-nt)	E-gel	17 ± 2.3%	28 ± 4.6%	49 ± 0.6%	35 ± 2.0%
	HPLC	6.5 ± 3.9%	24 ± 1.0%	46 ± 2.9%	38 ± 2.6%
CVB3-Factor VIII (7800-nt)	E-gel	11 ± 2.5%	28 ± 4.2%	49 ± 1.0%	23 ± 0.6%

Relative self-circularization efficiency before RNase R treatment was calculated from E-gel analysis as the percentage of the circRNA band intensity relative to the combined intensity of the circRNA and intact precursor bands. Relative self-circularization efficiencies determined by IP-RP HPLC analysis were estimated from the relative area of the circRNA peak. Values represent the mean ± SD from three independent experiments. Because E-gel electrophoresis and IP-RP HPLC differ in their analytical principles and quantification approaches, values should be compared only within each analytical method.

To determine whether the enhanced self-circularization efficiency affected the functionality of the resulting circRNAs, luciferase expression was compared between HPLC-purified CVB3-FLuc circRNAs generated using the conventional P1 and AS-polyA10-IGS P1 constructs. Comparable luciferase expression was observed between the two constructs (Supplementary Fig. S18), indicating that the optimized P1 design improves self-circularization efficiency without altering the protein expression of the resulting circRNA.

RNase R treatment substantially enriched the circRNA fraction generated by the optimized STS strategy by selectively removing residual linear RNA species, thereby facilitating subsequent purification. For the CVB3-Omicron spike construct, RNase R treatment increased the circRNA fraction to approximately 75% (Fig. 5B), and subsequent IP-RP HPLC purification yielded a circRNA preparation with >95% purity (Supplementary Fig. S19). These results demonstrate that STS-generated circRNAs can be efficiently enriched and purified to high purity for downstream applications.

## Discussion

In this study, we systematically investigated the effects of target site selection on the self-circularization efficiency of the end-to-end STS reaction. In addition, we developed P1 construct engineering strategies to further enhance the self-circularization efficiency of the STS reaction.

We observed that self-circularization efficiency varies substantially depending on the target site within the GOI, whether the same target sequence is placed at different positions or different target sequences are selected from within the GOI (Fig. 1). These results indicate that the success of the STS method depends on appropriate target site selection, as the resulting overall RNA structure shaped by target site-dependent GOI rearrangement in the P1 construct, together with accessibility of the target site to the IGS [25,27–30], could collectively influence ribozyme-mediated self-circularization efficiency. Therefore, extensive target site screening efforts may be necessary to identify an optimal target site for each GOI to achieve efficient self-circularization. In this regard, target site screening presents a practical approach to improve self-circularization efficiency across diverse GOIs. As an example, we identified the AU11 target site (5′-UUUAUU-3′) within the CVB3 IRES, which supported efficient self-circularization across various GOIs containing CVB3 IRES (Fig. 3).

The efficiency of STS-mediated self-circularization is likely influenced not only by the intrinsic reactivity of the ribozyme but also by the global folding of the precursor RNA. Complex RNA structures may either facilitate or hinder productive interactions between the IGS and the target site by affecting the accessibility of the target site and its productive interaction with the IGS. This structural constraint may become increasingly important as GOI length increases, potentially contributing to the reduced circularization efficiency often observed with long transcripts. In this context, target site selection and P1 construct engineering, including polyA10 and AS incorporation, may help overcome these structural constraints by promoting productive IGS-target site interactions. In addition to experimental identification of accessible uridines, target sites may also be predicted by computational approaches that calculate the binding free energies between candidate target sites and the IGS, providing a complementary strategy for target site selection and reducing the need for extensive experimental screening [31]. However, current RNA secondary structure prediction tools have limited accuracy for long RNAs, and the predicted structures were not always consistent with the experimentally observed self-circularization efficiencies. Therefore, although computational prediction may facilitate the design of P1 constructs and candidate target site selection, experimental validation remains essential. Future AI-based computational models may further improve target site prediction for STS-mediated self-circularization. Of note, the flexibility of the STS method in utilizing target sites with a 5′-NNNNNU-3′ sequence within the GOI enables the generation of scar-free circRNA even without requiring a spacer sequence (Fig. 3D), thereby allowing the production of circRNAs that are identical to their natural counterparts [20].

Engineering of the P1 construct further improved self-circularization efficiency when combined with target site screening. The polyA10-IGS P1 construct, in which the polyA10 sequence is introduced upstream of the IGS, enhanced efficiency across various GOIs, including long GOIs, compared with the conventional STS construct (Fig. 4, Table 1, and Supplementary Fig. S10). The sequence-dependent effects of the polyN region may partly reflect differences in the local structural environment surrounding the IGS (Supplementary Fig. S15). PolyA10 showed the highest self-circularization efficiency in both GOI contexts (CVB3-Omicron spike and CVB3-dCas9) and produced fewer by-product bands (Fig. 4) than the parental STS P1 (Supplementary Fig. S7). Poly(AG)5, which has relatively low predicted complementarity to itself and to neighboring sequences, also showed comparatively high and consistent efficiencies, whereas several sequences with greater potential for local base pairing showed lower efficiencies (Supplementary Fig. S15). PolyU10 may also engage in nonspecific base pairing with the A-rich region of the IGS. In the parental STS P1 construct, the three 5′ G residues introduced for T7 transcription are directly followed by the first G residue of the IGS, forming a stretch of four consecutive G residues. Insertion of polyA10 separates these G residues and may thereby alter the local RNA structure surrounding the IGS. By contrast, polyC10 may engage in nonspecific base pairing with the upstream GGG sequence, whereas the extended G-rich tract in the polyG10 construct may permit higher-order or intermolecular interactions, including potential G-quadruplex formation. However, this trend was not absolute, and some polyN sequences exhibited GOI-dependent effects (Supplementary Fig. S13). Collectively, these observations suggest that the polyN sequence may modulate potential base-pairing and other nonspecific interactions around the IGS. Although the precise mechanism by which the polyA sequence positioned upstream of the IGS enhances self-circularization remains unclear, gel analysis showed that the nonspecific bands observed with the original P1 construct (Supplementary Figs S7 and S14) were reduced following the introduction of polyA10 (Fig. 4). This finding suggests that polyA10, which has limited complementarity to neighboring sequences, may help maintain a more accessible local conformation, reduce nonspecific interactions, and thereby favor productive P1 formation, although the underlying mechanism remains to be investigated in greater detail in future studies.

Additional modification by introducing an AS into the polyA10-IGS P1 construct (AS-polyA10-IGS P1 construct), in which the AS is positioned upstream of the polyA10 to promote interaction between the IGS and the target site, further improved self-circularization efficiency (Fig. 5 and Table 1). Notably, this enhancement was particularly evident for long GOIs. For the CVB3-Factor VIII GOI (7.8 K-nt), the AS-polyA10-IGS P1 construct achieved ∼2-fold higher self-circularization efficiency than the PIE method, even without the need for a spacer sequence, highlighting a key advantage over PIE that relies on an intronic scar and an optimized spacer (AC108) for efficient circularization. Incorporation of the AS sequence may enhance specific IGS-target site interaction through complementary base pairing near the target site (Fig. 5A). Although the mechanism by which AS incorporation enhances self-circularization remains unclear, one possible explanation is that the AS-target interaction is transient and is subsequently replaced by the ribozyme-associated IGS. Once the target site is recruited in proximity to the ribozyme, the ribozyme-associated IGS may form a more favorable interaction with the target site, displacing the transient AS-target interaction and allowing formation of the catalytically productive IGS-target site duplex. Although this hypothesis remains to be experimentally validated, it may explain why AS incorporation enhances rather than inhibits STS-mediated self-circularization.

The AS-polyA10-IGS P1 construct strategy was optimized using the AU11 target site within the CVB3 IRES framework, and its performance may vary with other IRES elements or target site sequences. Therefore, additional optimization may be required when applying this strategy to different IRES contexts or alternative target sites. Furthermore, future studies evaluating the applicability of the STS strategy to other group I introns and identifying ribozymes with higher circularization efficiency will be of considerable interest, given the conserved P1-mediated target-recognition mechanism of group I introns.

In this study, we conducted a systematic evaluation of target site selection. In addition, P1 construct engineering further improved self-circularization efficiency. Together, these optimizations enhanced efficiency for long GOIs and, in the tested case, achieved higher performance than the conventional PIE method. The practical considerations and engineering strategies described here may support broader implementation of STS-based self-circularization across experimental and therapeutic applications. Overall, the optimized end-to-end STS approach provides an efficient platform for *in vitro* circRNA preparation without introducing extraneous sequences including spacers, thereby expanding its potential utility in biomedical applications.

## Supplementary Material

gkag869_Supplemental_File

## Data Availability

The data underlying this article are available in the article and in its online supplementary material.
