# Supplementary material for: Target site selection and P1 engineering enable highly efficient circular RNA production via end-to-end self-targeting and splicing
Source: Nucleic Acids Res. 2026 Sep 4;54(16):gkag869. doi: 10.1093/nar/gkag869 (PMC13542726; doi:10.1093/nar/gkag869)
Supplement: Supplementary file 1 [file gkag869_supplemental_file.pdf]

Supplementary Information

MANUSCRIPT TITLE

Target Site Selection and P1 Engineering Enable Highly Efficient Circular RNA Production via End-to-End Self-Targeting and Splicing

AUTHORS

Kyung Hyun Lee[1,*,†], Seongcheol Kim[1,†], Juwon Cha[1,2], and Seong-Wook Lee[1,2,*]

[1] R&D Center, Rznomics Inc., Seongnam 13468, Republic of Korea
[2] Department of Bioconvergence Engineering, Research Institute of Advanced Omics, Dankook University, Yongin 16890, Republic of Korea
* To whom correspondence should be addressed. Email: khlee@rznomics.com (K.H.L.); swl0208@dankook.ac.kr or swl0208@rznomics.com (S.-W.L)
† Kyung Hyun Lee and Seongcheol Kim contributed equally to this work.

Supplementary Table S1. Spacer and primer sequences used in this study. Yellow-highlighted sequence indicates the antisense sequences (AS) designed to bind the target site or its upstream region.

Spacer or Primer	Sequence (5′ to 3′)
**AC20 spacer**	AAAAAACAAAAAACAAAACA
**AC30 spacer**	ACAAAAAAAAAACCAAAAAAACAAAACACA
**AC40 spacer**	AAAAAACAAAAAACAAAACAAAAACAAAAAACAAAAAAAA
**AC70 spacer**	For sGFP:<br>AACCACAAAACACCCCAAACCAAAACAACCCCCAAAAAAAACCAAAAAAAACACAAAAAAAAACACACA<br><br>For RLuc:<br>AAAAAACAAAAAACAAAACGGCTATTATGCGTTACCGGCGCCGGAAACGCAATAGCCGAAAAACAAAAAA
**AC90 spacer**	AAAAAACAAAAAACAAAACGGCTATTATGCGTTACCGGCGCCGGAAACGCAATAGCCGAAAAACAAAAAACAAAAAAAACAAAAAAAAAA
**AC108 spacer**	AAAAAACAAAAAACAAAACGGCTATTATGCGTTACCGGCGCCGGAAACGCAATAGCCGAAAAACAAAAAACAAAAAAAACAAAAAAAAAACCAAAAAAACAAAACACA (Underlined: Target site)
**F Primer for 3′-end target sequencing (Seq F1)**	AGGATGGCAGCGTGCAGCTGGCTGA
**R Primer for 3′-end target sequencing (Seq R1)**	GTCCGGGGTAACAGAAGTGCTTGAT
**F Primer for IRES target sequencing (Seq F2)**	CACACACCGATCAACAGTCAGCGTG
**R Primer for IRES target sequencing (Seq R2)**	GCTCTATTAGTCACCGGATGGCCAA
**F Primer for target gene target sequencing (Seq F3)**	GGCGAGGGTGAAGGTGACGC
**R Primer for target gene target sequencing (Seq R3)**	CGTTCGGATCTTTAGACAGAACGCT
**F Primer for IRES target sequencing 2 (Seq F4)**	ACAGACATGGTGCGAAGAGTCTATT
**R Primer for IRES target sequencing 2 (Seq R4)**	AGGTACCGGCAGTTTACCAGTAGTA
**T7 AU11 polyA10 F primer (P1)**	ATAATACGACTCACTATAGGGAAAAAAAAAAGATAAAAAAAAGTTATCAGGCATGCACCTGGT
**T7 AU11 polyT10 F primer (P1)**	ATAATACGACTCACTATAGGGTTTTTTTTTTGATAAAAAAAAGTTATCAGGCATGCACCTGGT

**T7 AU11 polyG10 F primer (P1)**	ATAATACGACTCACTATAGGG<u>GGGGGGGGGG</u>GATAAAAAAAGTTATCAGGCATGCACCTGGT
**T7 AU11 polyC10 F primer (P1)**	ATAATACGACTCACTATAGGG<u>CCCCCCCCCC</u>GATAAAAAAAGTTATCAGGCATGCACCTGGT
**T7 AU19 polyA10 F primer (P1)**	ATAATACGACTCACTATAGGG<u>AAAAAAAAAA</u>GTAATAAAAAGTTATCAGGCATGCACCTGGT
**T7 Spacer Target polyA10 F primer (P1)**	ATAATACGACTCACTATAGGG<u>AAAAAAAAAA</u>GGCCGTAAAAGTTATCAGGCATGCACCTGGT
**T7 AU11 AS6-polyA10-IGS F primer (P1)**	ATAATACGACTCACTATAGG<u>GATAAA</u>AAAAAAAAAAGATAAAAAAAGTTATCAGGCATGCACCTGGT
**T7 AU11 AS8 (No IGS)-polyA10-IGS F primer (P1)**	ATAATACGACTCACTATAGGGATGAAACAAAAAAAAAAAGATAAAAAAAGTTATCAGGCATGCACCTGGT
**T7 AU11 AS14-polyA10-IGS F primer (P1)**	ATAATACGACTCACTATAGG<u>GATAAA</u>ATGAAACAAAAAAAAAAAGATAAAAAAAGTTATCAGGCATGCACCTGGT

A

CVB3 IRES

TTAAAACAGCCTGTGGGTTGATCCCACCCACAGGCCCATTGGGCGCTAGCACTCTGGTATCACGGTA
CCTTTGTGCGCCTGTTTTATACCCCCTCCCCCAACTGTAACTTAGAAGTAACACACACCGATCAACAG
TCAGCGTGGCACACCAGCCACGTTTTGATCAAGCACTTCTGTTACCCCGGACTGAGTATCAATAGAC
TGCTCACGCGGTTGAAGGAGAAAGCGTTCGTTATCCGGCCAACTACTTCGAAAAACCTAGTAACACC
↑ AU16 target site addition
GTGGAAGTTGCAGAGTGTTTCGCTCAGCACTACCCCAGTGTAGATCAGGTCGATGAGTCACCGCATT
Target site (IRES 2)
CCCCACGGGCGACCGTGGCGGTGGCTGCGTTGGCGGCCTGCCCATGGGGAAACCCATGGGACGCT
CTAATACAGACATGGTGCGAAGAGTCTATTGAGCTAGTTGGTAGTCCTCCGGCCCCTGAATGCGGCTA
ATCCTAACTGCGGAGCACACACCCTCAAGCCAGAGGGCAGTGTGTCGTAACGGGCAACTCTGCAGC
GGAACCGACTACTTTGGGTGTCCGTGTTTCATTTTATTCCTATACTGGCTGCTTATGGTGACAATTGAG
AU11 target site
AU28 target site (IRES 1)
AGATCGTTACCATATAGCTATTGGATTGGCCATCCGGTGACTAATAGAGCTATTATATATCCCTTTGTTG
AU19 target site
GGTTTATACCACTTAGCTTGAAAGAGGTTAAAACATTACAATTCATTGTTAAGTTGAATACAGCAAA

B

sGFP

ATGCGTAAAGGCGAAGAGCTGTTCACTGGTGTCGTCCCTATTCTGGTGGAACTGGATGGTGATGTCA
ACGGTCATAAGTTTTCCGTGCGTGGCGAGGGTGAAGGTGACGCAACTAATGGTAAACTGACGCTGA
AGTTCATCTGTACTACTGGTAAACTGCCGGTACCTTGGCCGACTCTGGTAACGACGCTGACTTATGGT
GTTCAGTGCTTTGCTCGTTATCCGGACCATATGAAGCAGCATGACTTCTTCAAGTCCGCCATGCCGGA
AGGCTATGTGCAGGAACGCACGATTTCCTTTAAGGATGACGGCACGTACAAAACGCGTGCGGAAGT
GAAATTTGAAGGCGATACCCTGGTAAACCGCATTGAGCTGAAAGGCATTGACTTTAAAGAAGACGG
AU32 target site
↑ AU16 target site addition
CAATATCCTGGGCCATAAGCTGGAATACAATTTTAACAGCCACAATGTTTACATCACCGCCGATAAAC
AAAAAAATGGCATTAAAGCGAATTTTAAAATTCGCCACAACGTGGAGGATGGCAGCGTGCAGCTGG
CTGATCACTACCAGCAAAACACTCCAATCGGTGATGGTCCTGTTCTGCTGCCAGACAATCACTATCTG
AGCACGCAAAGCGTTCTGTCTAAAGATCCGAACGAGAAACGCGATCATATGGTTCTGCTGGAGTTCG
TAACCGCAGCGGGCATCACGCATGGTATGGATGAACTGTACAAA
↑ AU16 target site addition (3′ end)

Supplementary Figure S1. (**A**) Sequences and target site positions within the CVB3 IRES. (**B**) Sequences and target site positions within the sGFP CDS. For AU16 target site, the target sequence was introduced at each position indicated by the arrows.

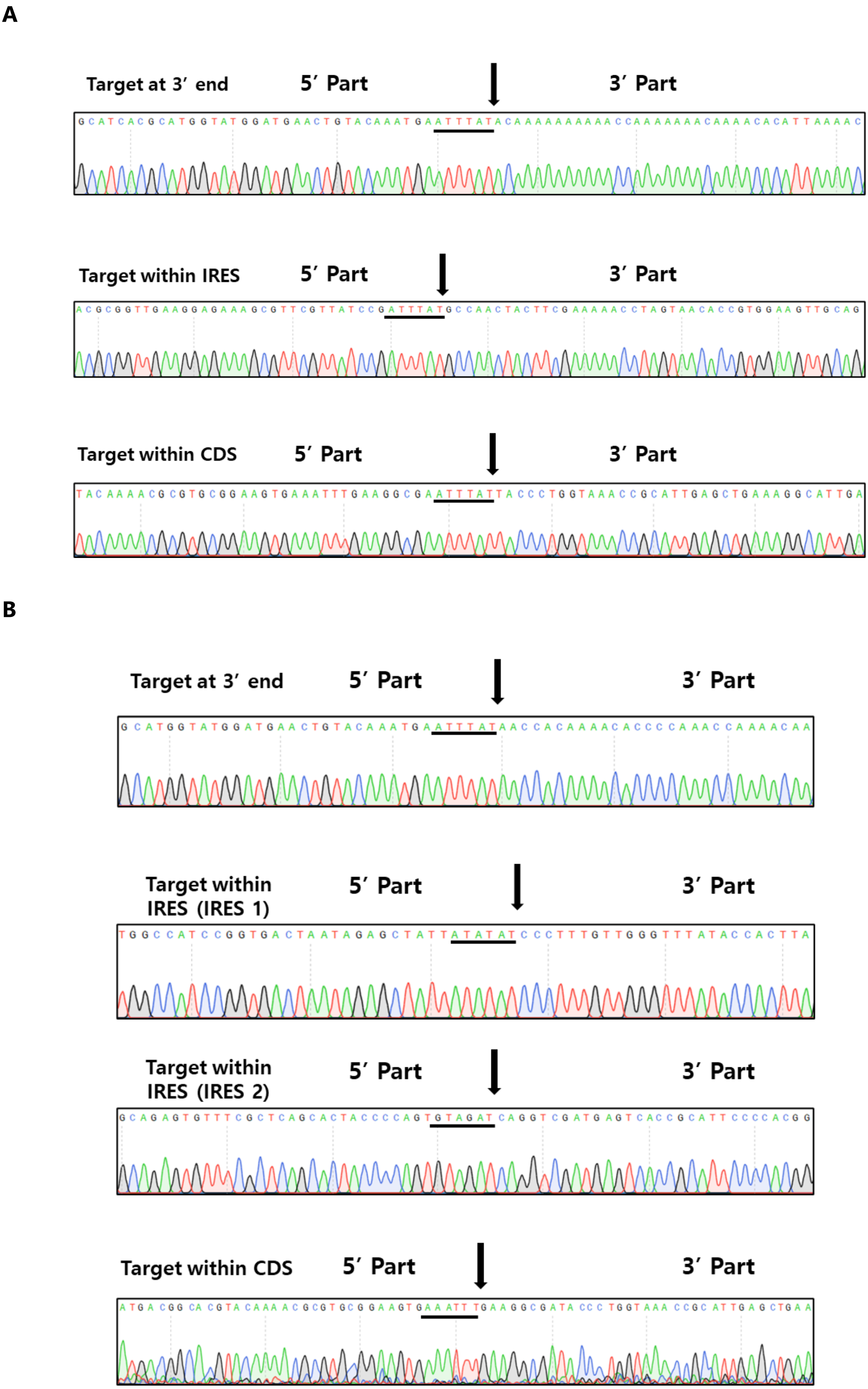
A
Target at 3′ end
5′ Part
3′ Part
Target within IRES
5′ Part
3′ Part
Target within CDS
5′ Part
3′ Part
B
Target at 3′ end
5′ Part
3′ Part
Target within IRES (IRES 1)
5′ Part
3′ Part
Target within IRES (IRES 2)
5′ Part
3′ Part
Target within CDS
5′ Part
3′ Part

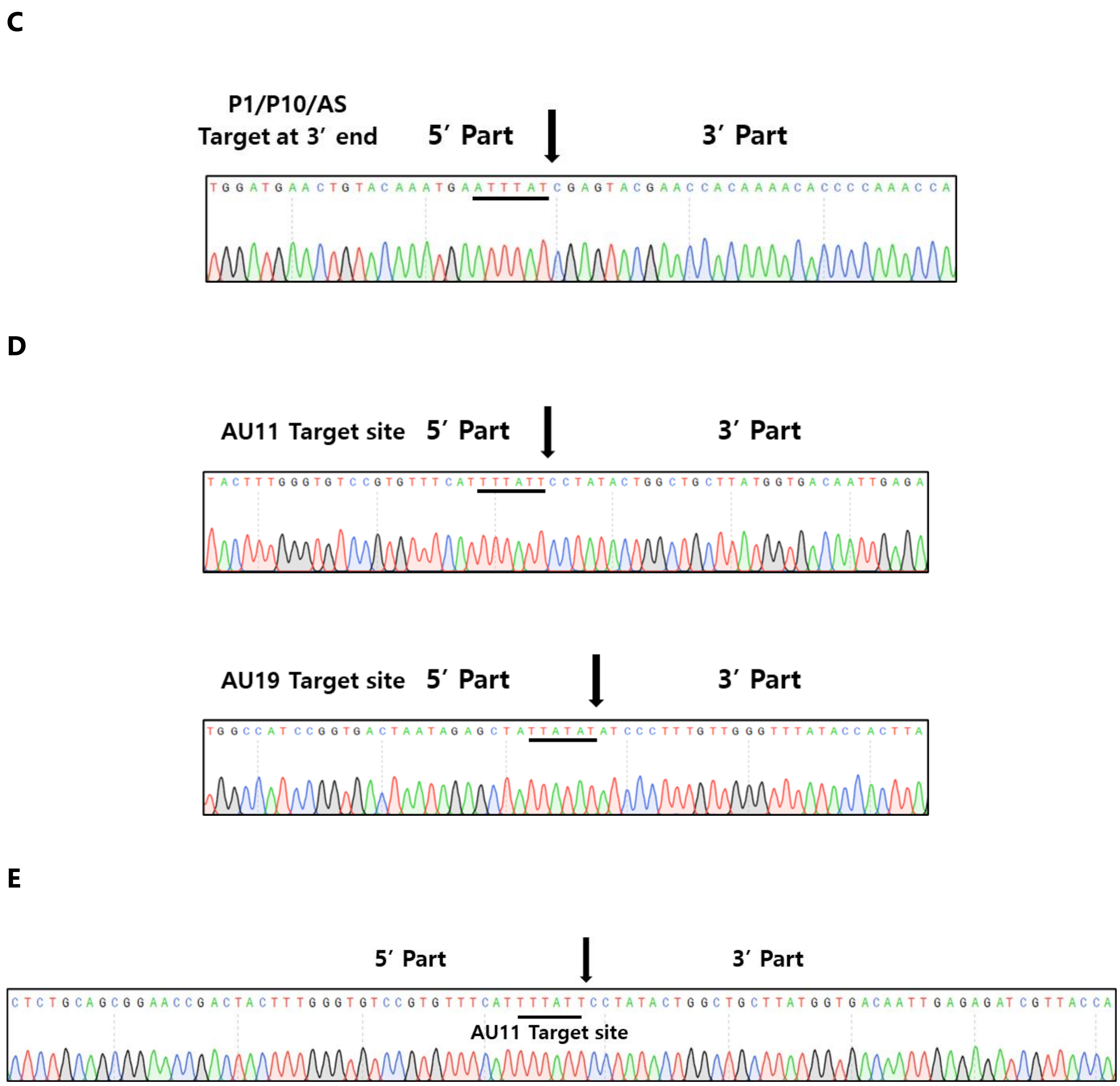
C
P1/P10/AS
Target at 3′ end
5′ Part
3′ Part
D
AU11 Target site
5′ Part
3′ Part
AU19 Target site
5′ Part
3′ Part
E
5′ Part
3′ Part
AU11 Target site

F

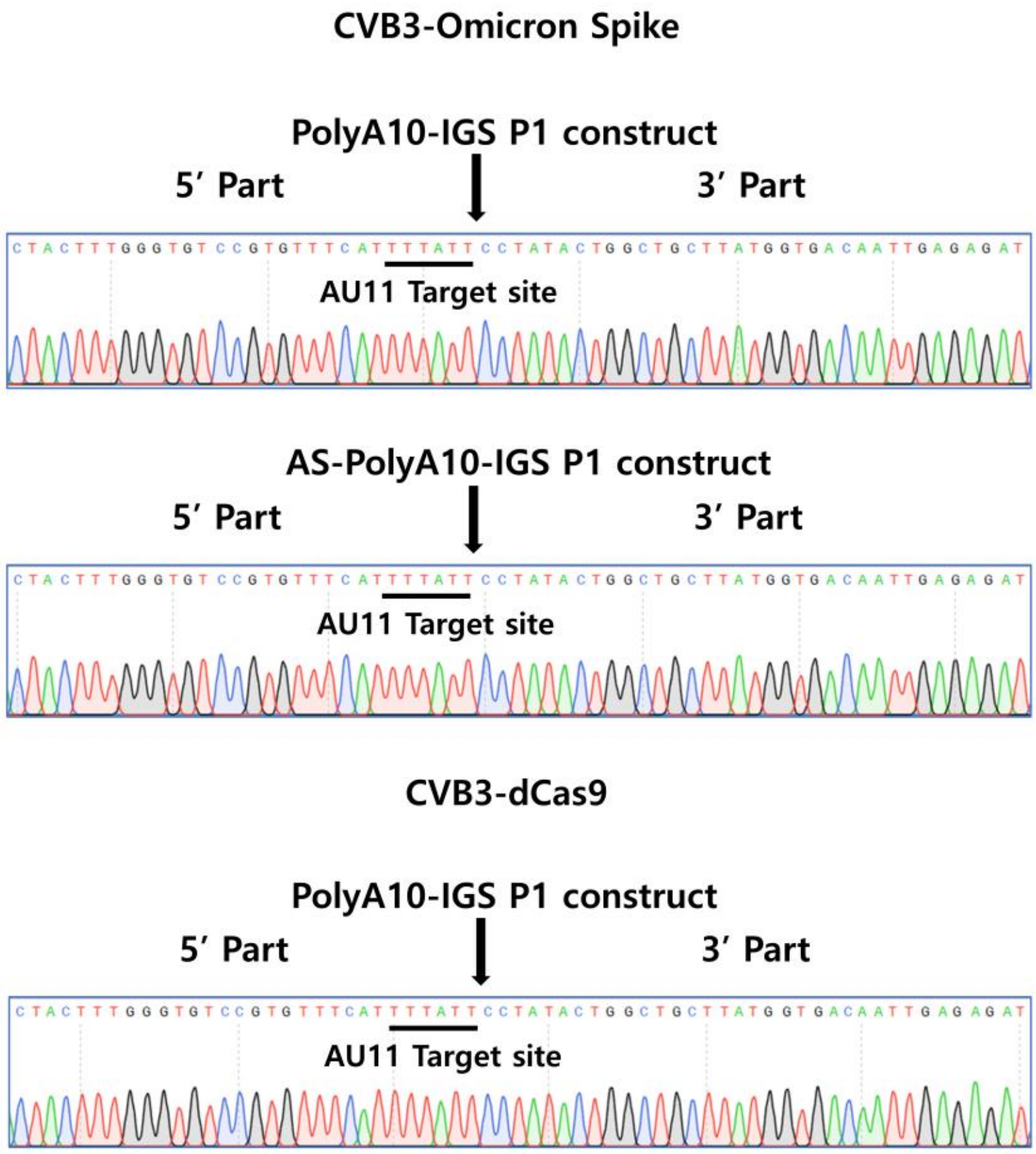

CVB3-Omicron Spike
PolyA10-IGS P1 construct
5′ Part
3′ Part
AU11 Target site
AS-PolyA10-IGS P1 construct
5′ Part
3′ Part
AU11 Target site
CVB3-dCas9
PolyA10-IGS P1 construct
5′ Part
3′ Part
AU11 Target site

G

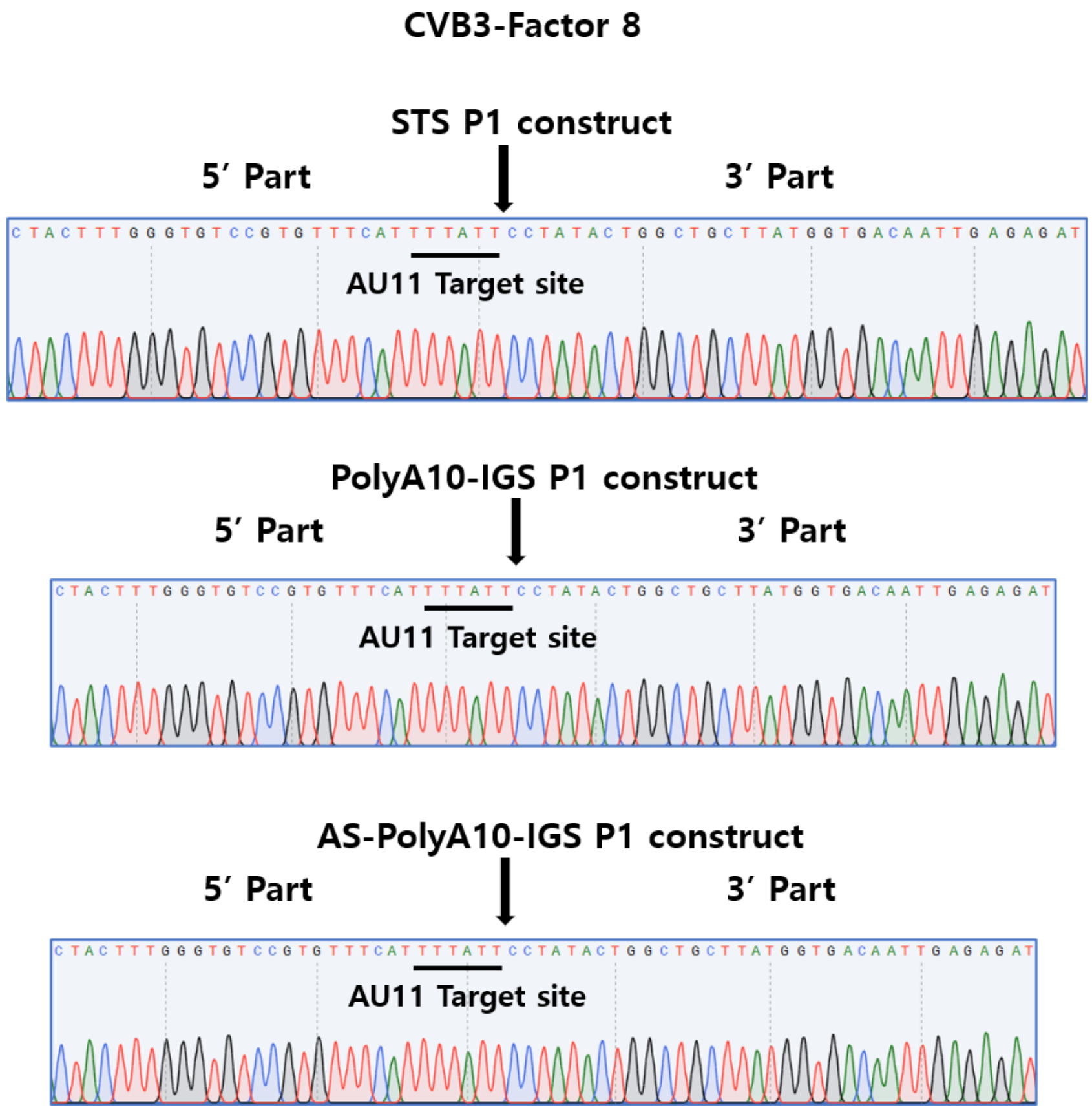

Supplementary Figure S2. Precise STS-mediated circRNA generation for each construct was confirmed by sequencing analysis of the junction region. (**A**) Sequencing analysis results of P1 constructs for the CVB3-sGFP-AC30 GOI with the AU16 target sequence at different positions. (**B**) Sequencing analysis results of P1 constructs for the CVB3-sGFP-AC70 GOI with various target sequences at different positions. (**C**) Sequencing analysis result of P1/P10/AS construct for CVB3-sGFP-AC70 GOI using the AU16 target site at the 3'-end of the CDS. (**D**) Sequencing analysis results of P1 constructs for the CVB3-sGFP-AC40 GOI using either the AU11 or AU19 target site. (**E**) Sequencing analysis result of P1 construct for the CVB3-RLuc mutant-AC0 GOI using the AU11 target site, shown as a representative example. (**F**) Sequencing analysis results of PolyA10-IGS P1 or AS-polyA10-IGS constructs for the CVB3-Omicron spike protein GOI, and those of PolyA10-IGS P1 construct for the CVB3-dCas9 GOI, using the AU11 target site. (**G**) Sequencing analysis results of P1, PolyA10-IGS P1, or AS-polyA10-IGS constructs for the CVB3-Factor VIII GOI using the AU11 target site. Target sequences are underlined. The specific splicing junction is indicated by an arrow.

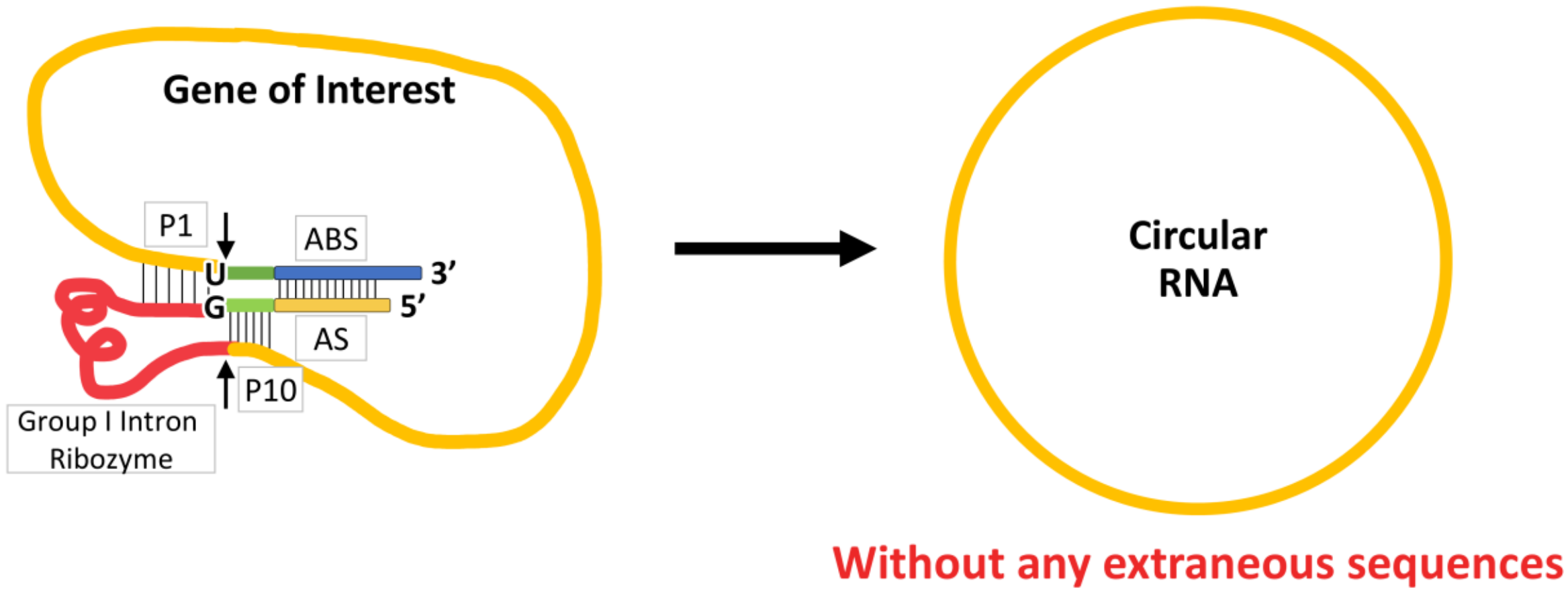

Supplementary Figure S3. Schematic of P1/P10/AS construct used for the CVB3-sGFP-AC70 GOI using the AU16 target site at the 3'-end of the CDS. STS reaction occurs via two consecutive transesterification reactions, through which the regions indicated by arrows are joined.

A

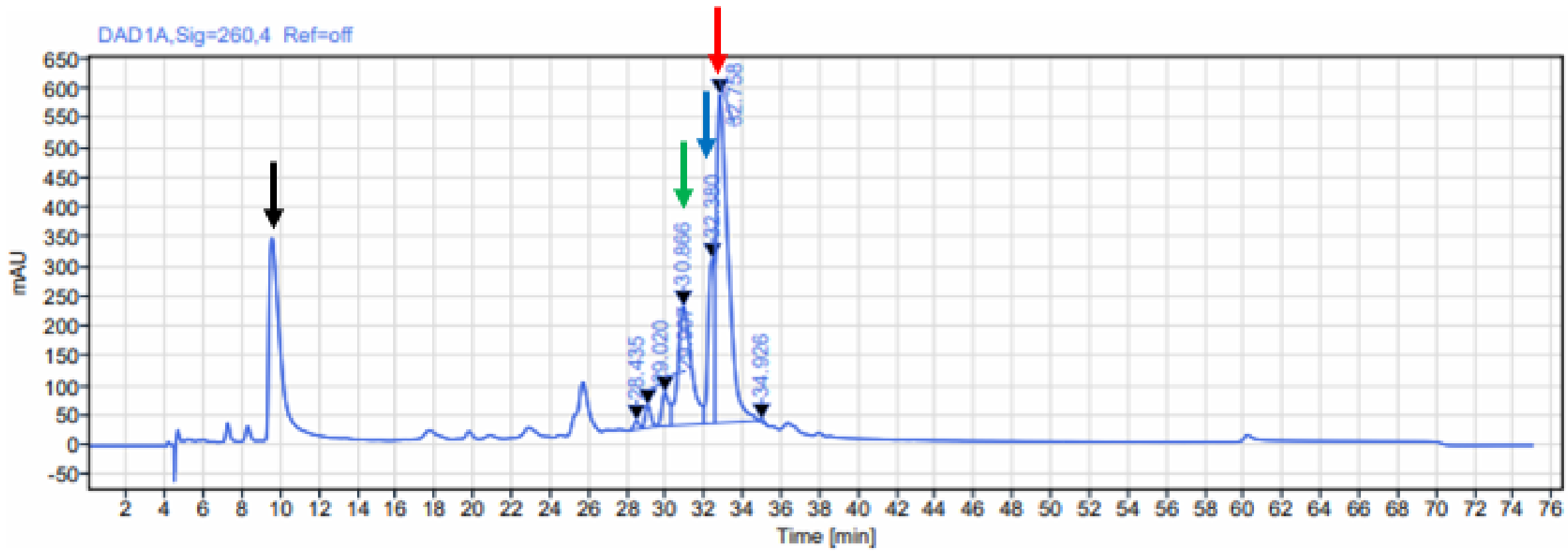

Signal: DAD1A,Sig=260,4 Ref=off

RT [min]	Type	Width [min]	Area	Height	Area%	Name
28.435	BV	0.66	263.55	14.21	0.63	
29.020	VB	0.75	756.77	37.53	1.81	
29.907	BV	0.77	1491.83	56.14	3.57	
30.866	VV	1.68	9626.23	198.87	23.06	
32.380	VV	0.57	5828.27	275.76	13.96	
32.758	VB	2.29	23695.33	551.37	56.77	
34.926	BB	0.48	75.78	4.57	0.18	
		**Sum**	**41737.76**			

B

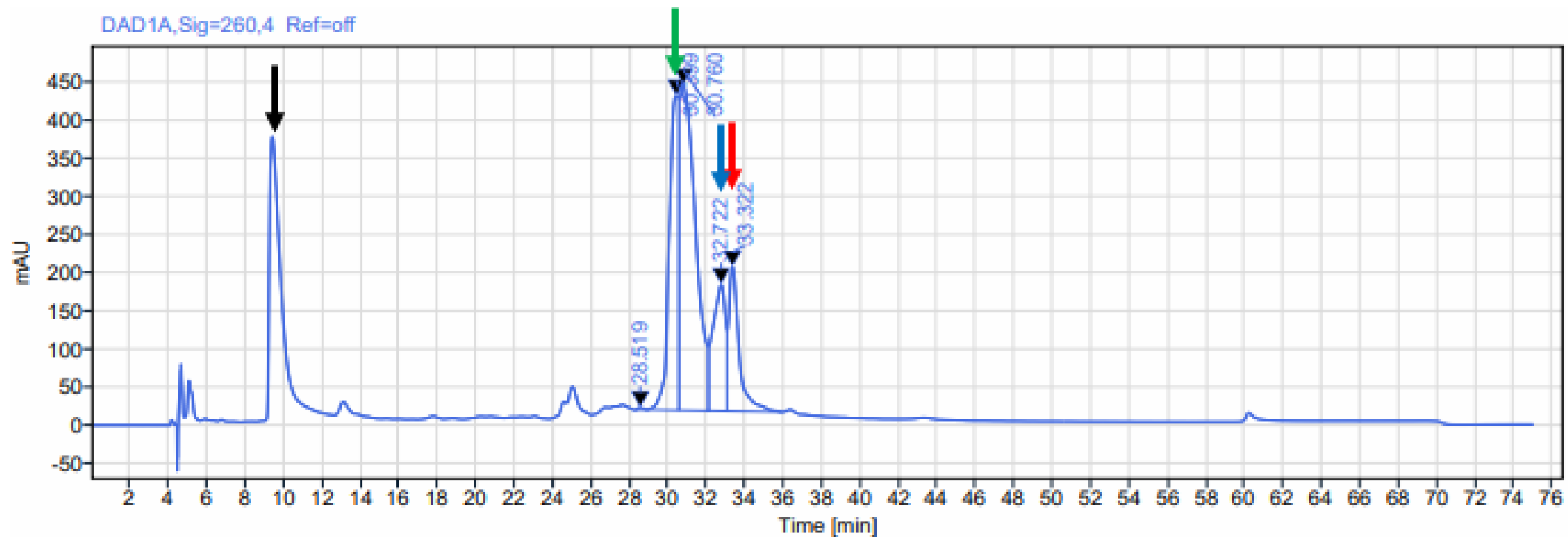

Signal: DAD1A,Sig=260,4 Ref=off

RT [min]	Type	Width [min]	Area	Height	Area%	Name
28.519	BB	0.62	54.00	3.01	0.10	
30.399	BV	1.69	14096.72	413.74	26.29	
30.760	VV	1.52	24631.90	428.21	45.93	
32.722	VV	0.99	7669.70	166.60	14.30	
33.322	VB	2.85	7177.90	189.72	13.38	
		**Sum**	**53630.22**			

C

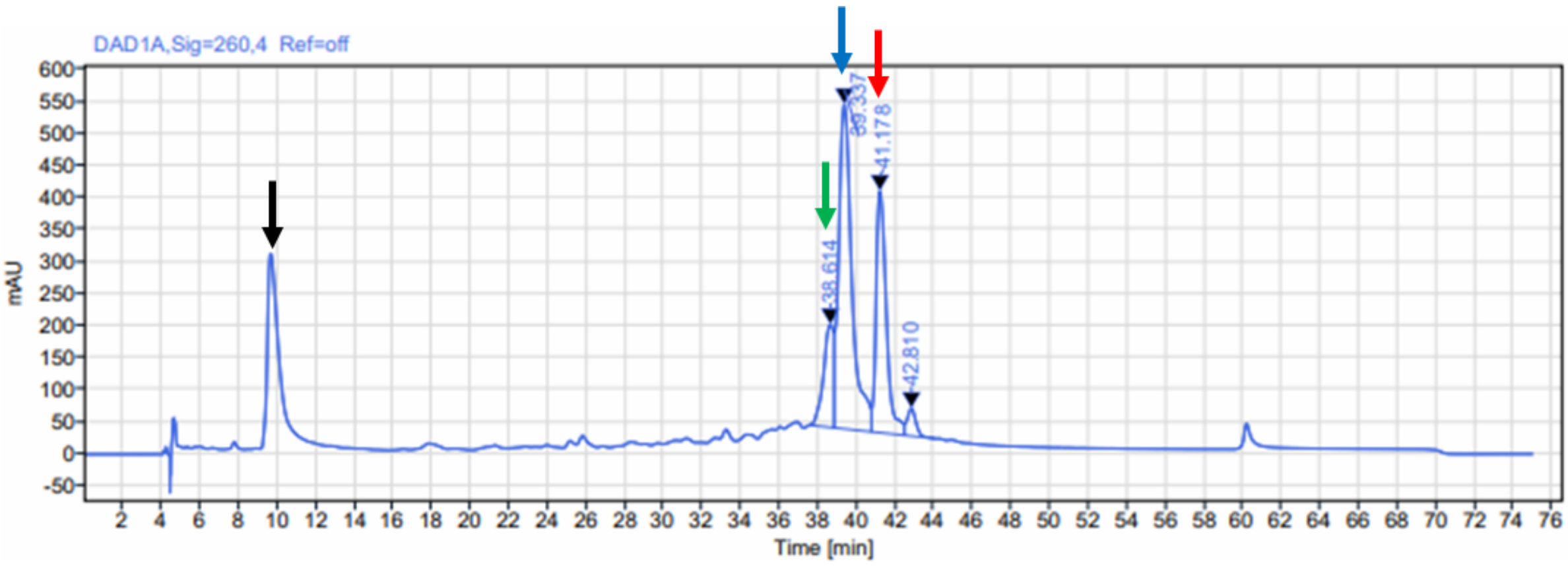

Signal:	DAD1A,Sig=260,4 Ref=off					
**RT [min]**	**Type**	**Width [min]**	**Area**	**Height**	**Area%**	**Name**
38.614	BV	1.29	5732.98	160.26	12.62	
39.337	VV	1.93	24344.79	506.40	53.61	
41.178	VV	1.70	13960.57	377.11	30.74	
42.810	VBA	1.56	1376.67	43.22	3.03	
		**Sum**	**45415.01**			

D

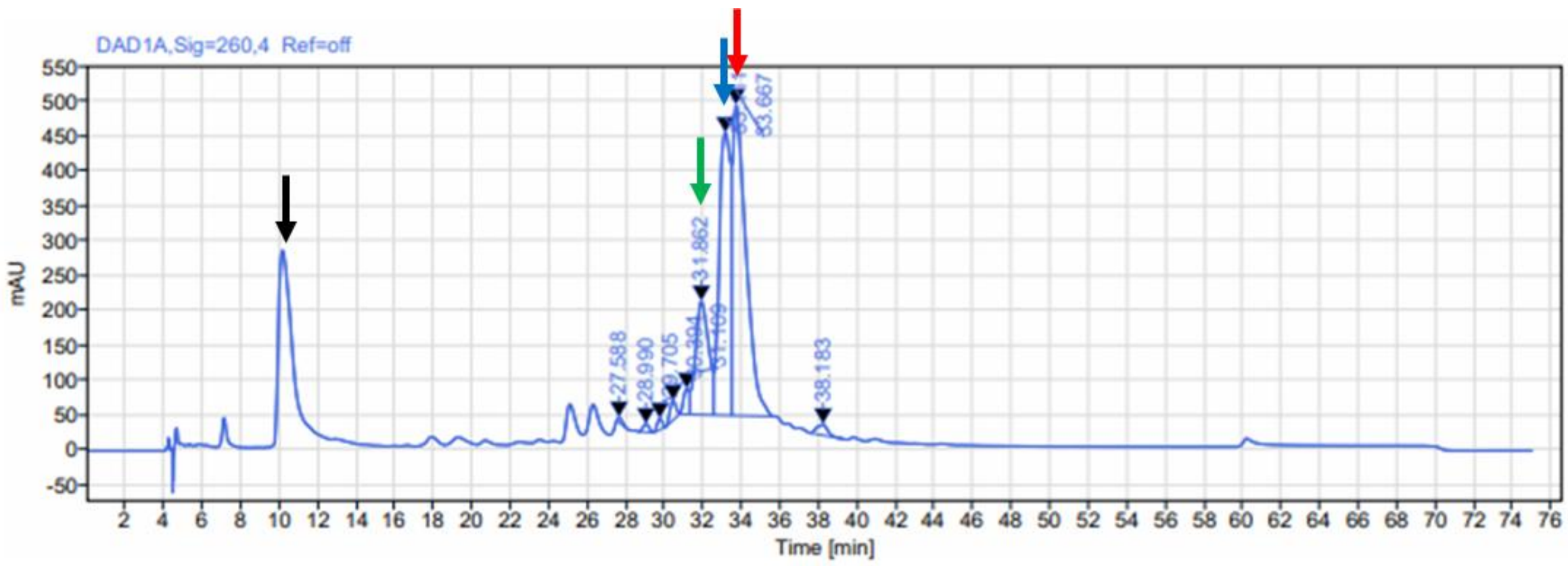

Signal:	DAD1A,Sig=260,4 Ref=off					
**RT [min]**	**Type**	**Width [min]**	**Area**	**Height**	**Area%**	**Name**
27.588	BB	0.53	116.94	6.61	0.24	
28.990	BB	0.75	250.60	11.38	0.52	
29.705	BB	0.62	291.04	15.52	0.60	
30.394	BB	0.70	605.73	27.62	1.25	
31.109	BV	0.53	734.30	36.16	1.52	
31.862	VV	1.24	7614.42	161.94	15.75	
33.111	VV	0.94	16507.11	403.50	34.15	
33.667	VB	2.18	21615.03	444.07	44.72	
38.183	BB	1.72	604.20	15.73	1.25	
		**Sum**	**48339.38**			

E

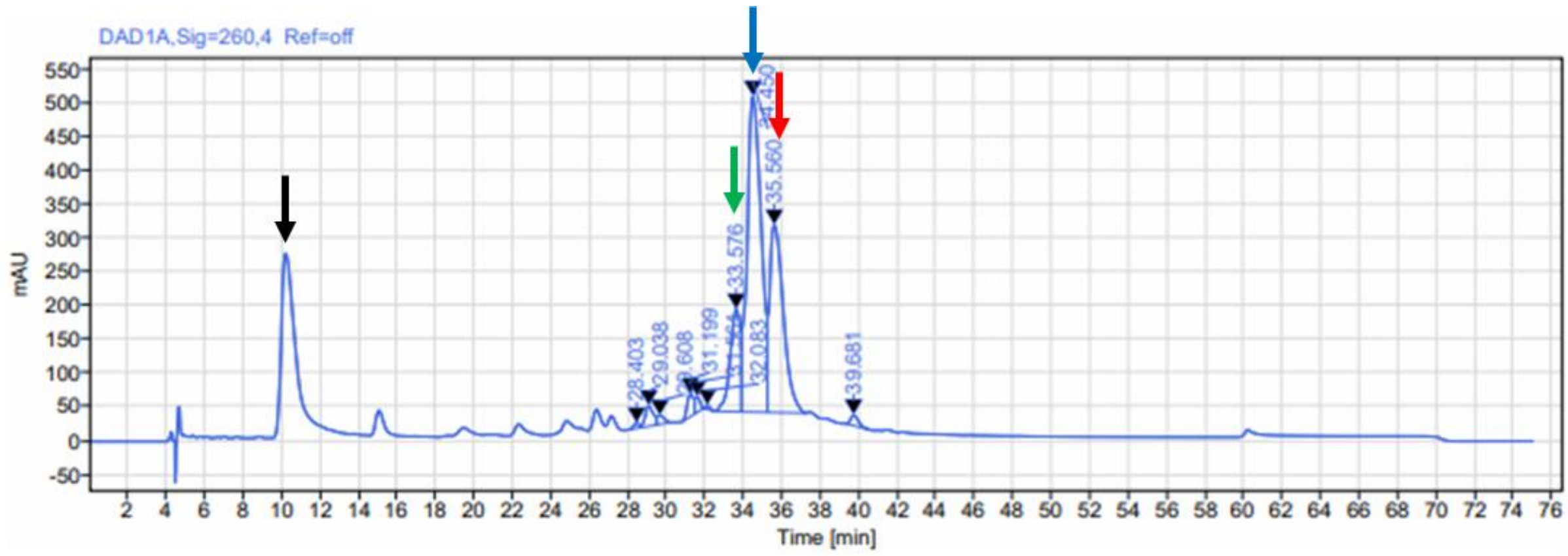

Signal: DAD1A,Sig=260,4 Ref=off

RT [min]	Type	Width [min]	Area	Height	Area%	Name
28.403	BV	0.64	149.96	7.31	0.32	
29.038	VV	0.81	870.62	29.82	1.88	
29.608	VB	0.74	338.87	12.71	0.73	
31.199	BV	0.63	766.40	33.44	1.66	
31.561	VB	0.47	410.66	22.59	0.89	
32.083	BB	0.52	89.37	5.72	0.19	
33.576	BV	1.40	5598.34	151.31	12.10	
34.450	VV	1.35	23744.94	466.81	51.34	
35.560	VB	1.99	13858.45	276.87	29.96	
39.681	BB	1.31	424.24	15.29	0.92	
		**Sum**	**46251.85**			

Supplementary Figure S4. HPLC-based self-circularization efficiency analysis using peak area integration. Circularization efficiency was calculated as the percentage of circRNA peak area relative to total peak area within the defined peak region, excluding cleaved ribozyme peaks. STS reaction was performed using the P1 construct. (**A**) GOI: CVB3-sGFP (1498-nt) using the AU11 target site. (**B**) GOI: CVB3-sGFP using the AU19 target site. (**C**) GOI: CVB3-FLuc (2434-nt) using the AU11 target site. (**D**) GOI: CVB3-Gluc (1339-nt) using the AU11 target site. (**E**) GOI: CVB3-EPO (1363-nt) using the AU11 target site. Black, green, blue, and red arrows indicate the cleaved group I intron ribozyme, self-cleaved RNA, intact precursor, and circRNA, respectively. The red box indicates the circRNA peak area.

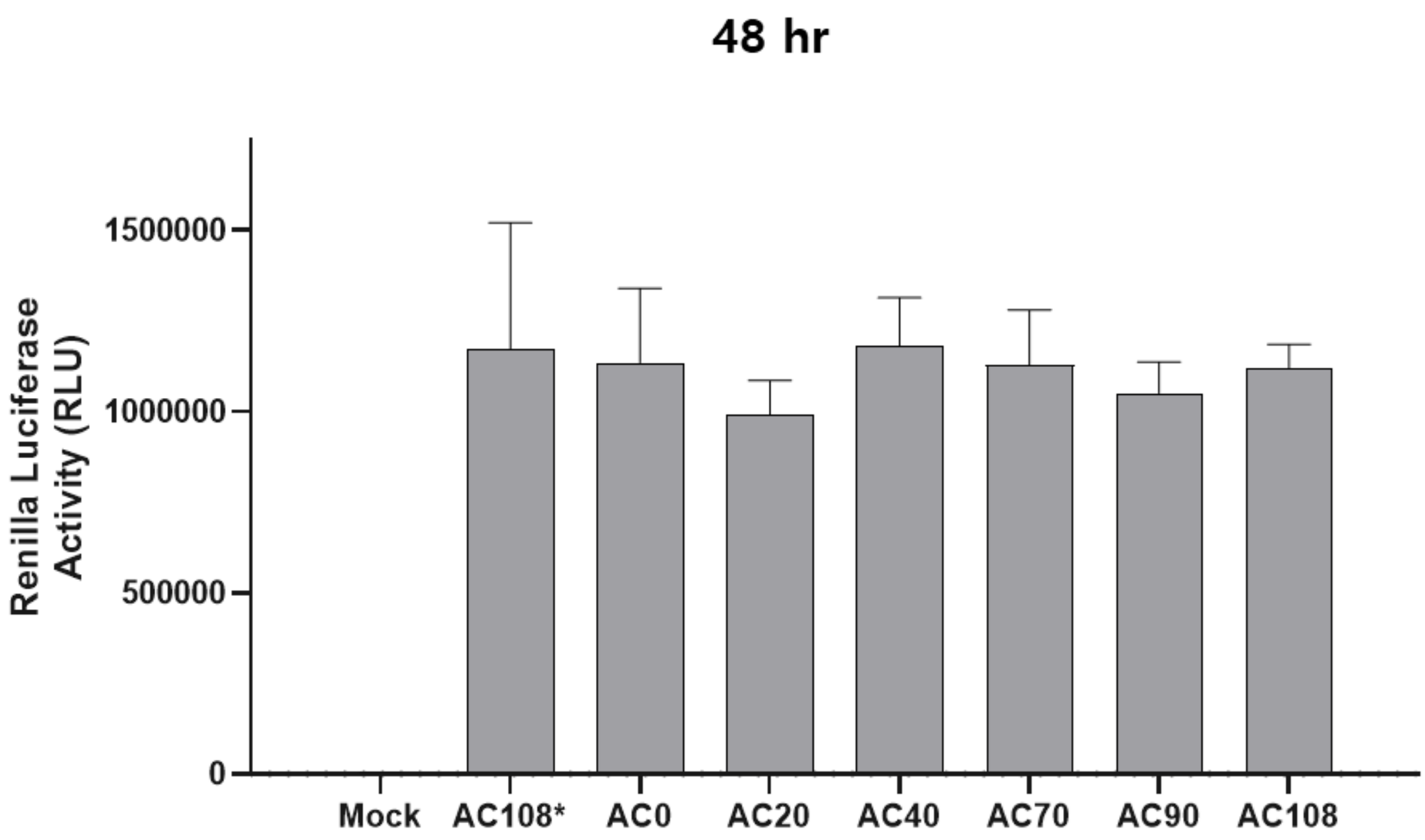

Supplementary Figure S5. Reporter protein expression from CVB3-RLuc mutant circRNAs using the AU11 target site within the CVB3 IRES, containing spacer sequences of varying lengths in HEK293A cells. Renilla luciferase activity was measured 48 hours post-transfection. The asterisk indicates the construct using the spacer target site for the STS reaction.

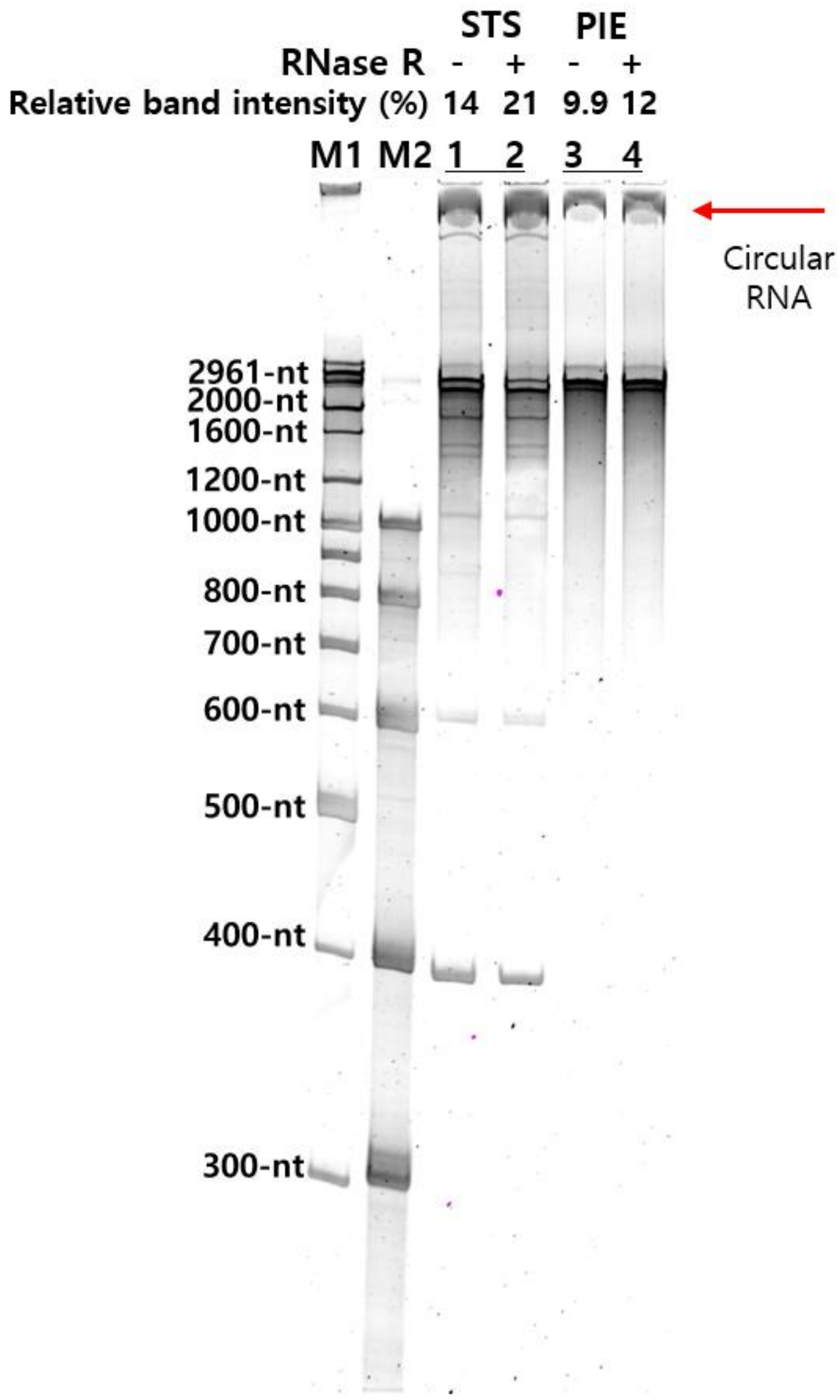

Supplementary Figure S6. Comparison of self-circularization efficiency between STS and PIE methods using a spacer-free CVB3-FLuc GOI. 4% denaturing PAGE analysis of IVT samples (lane 1 for STS and lane 3 for PIE) or RNase R-treated IVT samples (lane 2 for STS and lane 4 for PIE), comparing the relative band intensity of circRNA generated by P1 constructs using STS and PIE methods. The CVB3–FLuc GOI (2397 nt) does not contain a spacer.

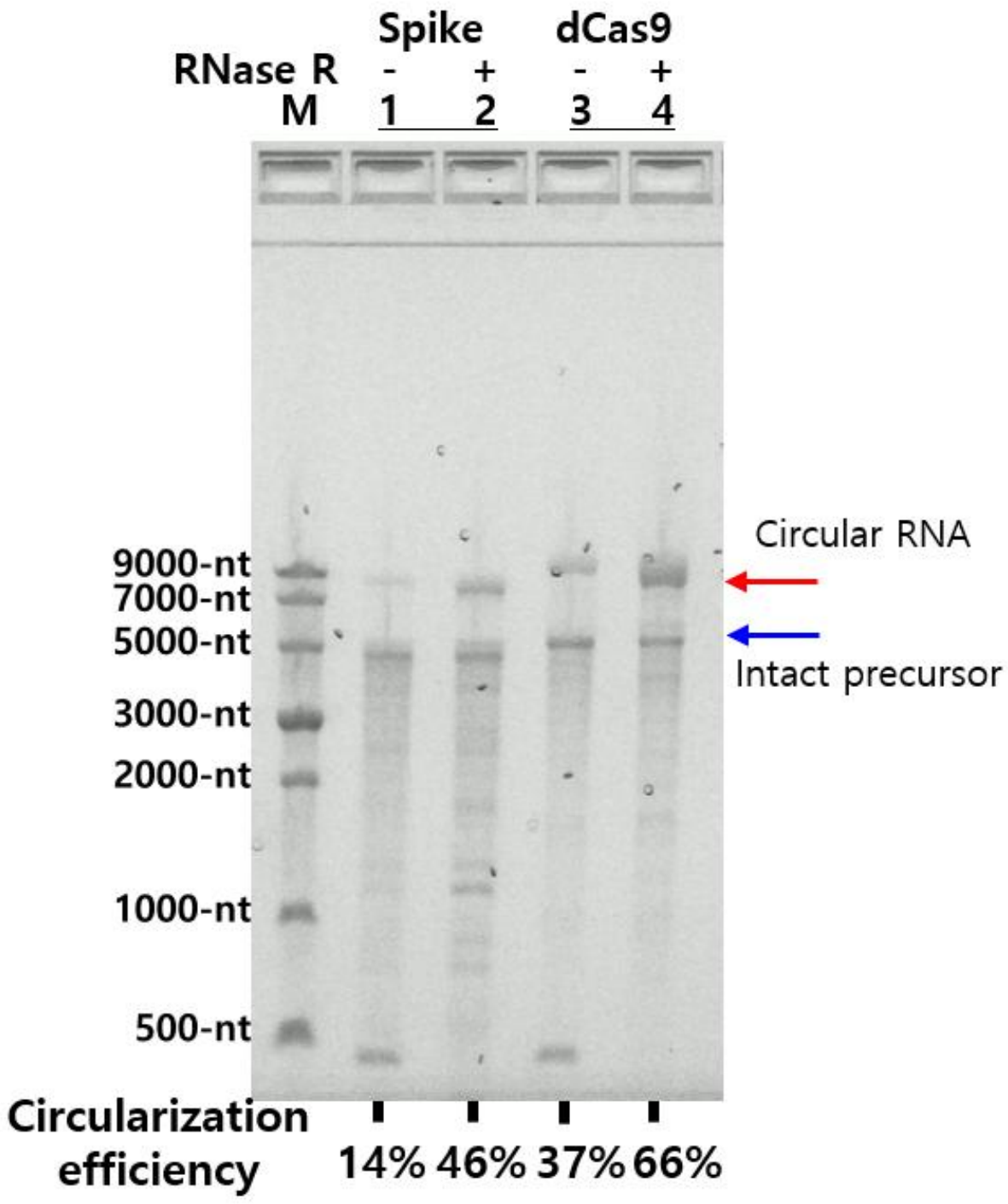

Supplementary Figure S7. 1% E-gel EX agarose gel analysis of IVT samples or RNase R-treated IVT samples from original P1 constructs generating CVB3-Omicron spike or CVB3-dCas9 circRNAs, respectively. CVB3-Spike and CVB3-dCas9 IVT samples (lane 1 and 3) or RNase R-treated IVT samples (lane 2 and lane 4). Circularization efficiency was determined based on relative circRNA band intensity.

A

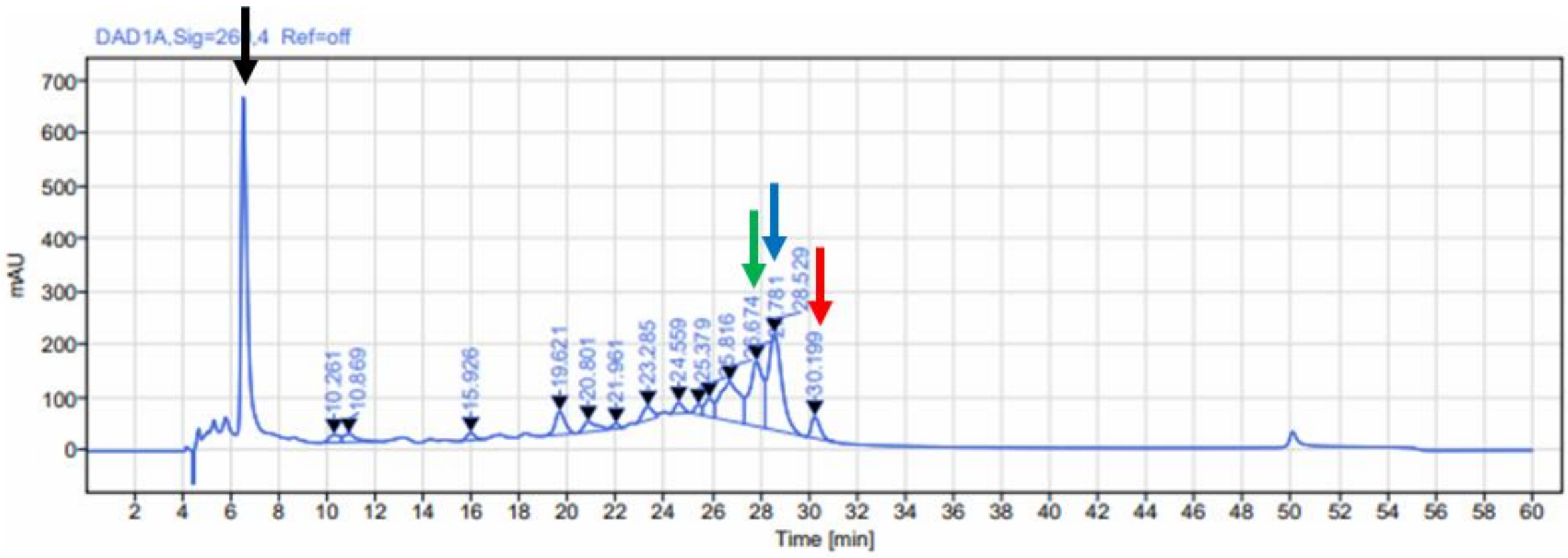

Signal:	DAD1A,Sig=260,4 Ref=off					
**RT [min]**	**Type**	**Width [min]**	**Area**	**Height**	**Area%**	**Name**
10.261	BV	0.93	439.63	14.35	1.92	
10.869	VB	1.29	547.98	15.03	2.40	
15.926	BB	0.96	318.92	13.51	1.39	
19.621	BB	1.39	1256.99	44.18	5.50	
20.801	BV	1.30	734.53	19.41	3.21	
21.961	VB	0.65	196.93	8.88	0.86	
23.285	BV	0.88	751.96	25.33	3.29	
24.559	BB	0.86	461.34	20.15	2.02	
25.379	BV	0.49	347.21	18.10	1.52	
25.816	VV	0.51	874.02	34.38	3.82	
26.674	VV	1.24	4166.28	74.05	18.22	
27.781	VV	0.87	4661.87	122.94	20.39	
28.529	VB	1.60	7091.52	182.26	31.01	
30.199	BB	1.36	1016.85	41.30	4.45	
		**Sum**	**22866.03**			

B

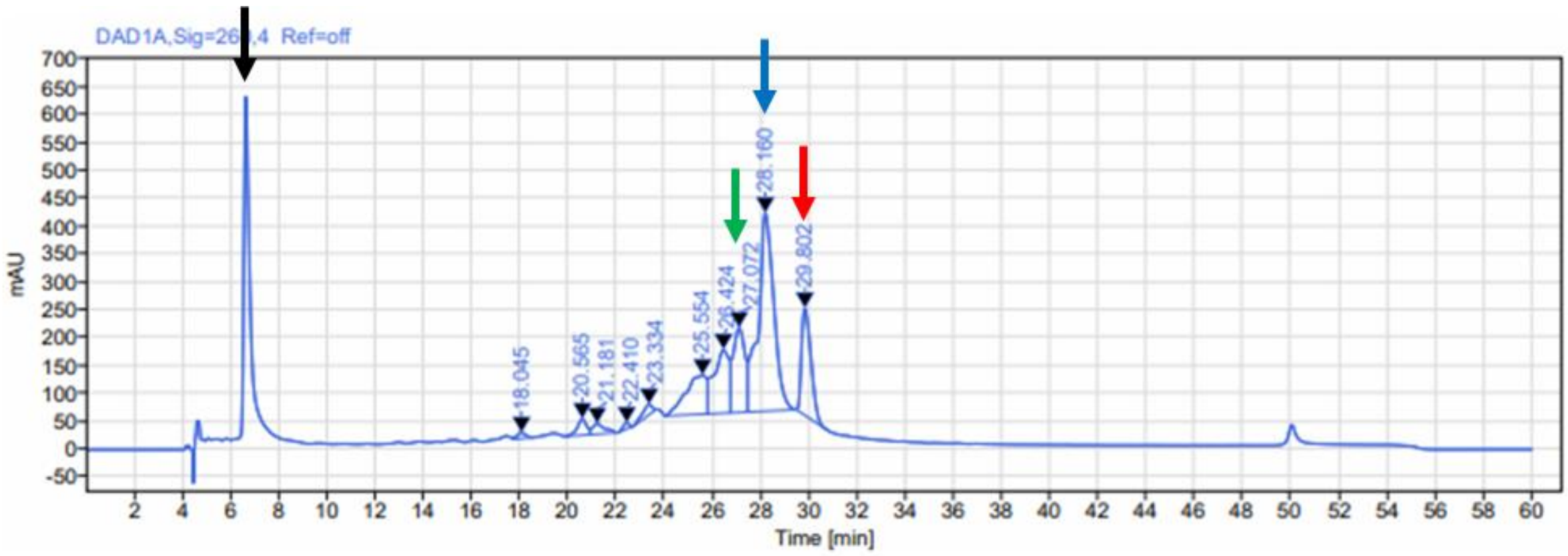

Signal: DAD1A,Sig=260,4 Ref=off

RT [min]	Type	Width [min]	Area	Height	Area%	Name
18.045	VB	0.81	250.04	10.06	0.65	
20.565	BV	1.01	774.55	28.88	2.02	
21.181	VB	1.13	674.89	18.49	1.76	
22.410	BB	0.60	193.81	10.26	0.51	
23.334	BB	0.93	526.83	17.90	1.38	
25.554	BV	1.74	4246.53	69.57	11.09	
26.424	VV	0.95	5009.14	113.31	13.08	
27.072	VV	0.71	5188.96	151.19	13.55	
28.160	VB	2.02	16174.73	354.81	42.22	
29.802	BB	1.29	5267.06	191.59	13.75	
		**Sum**	**38306.55**			

Supplementary Figure S8. HPLC-based self-circularization efficiency analysis using peak area integration. Circularization efficiency was calculated as the percentage of circRNA peak area relative to the total peak area within the defined peak region, excluding cleaved ribozyme peaks. STS reaction was performed using the P1 construct with the AU11 target site. (**A**) GOI: CVB3-Omicron spike protein (4569-nt). (**B**) GOI: CVB3-dCas9 (4851-nt). Black, green, blue, and red arrows indicate the cleaved group I intron ribozyme, self-cleaved RNA, intact precursor, and circRNA, respectively. The red box indicates the circRNA peak area.

A

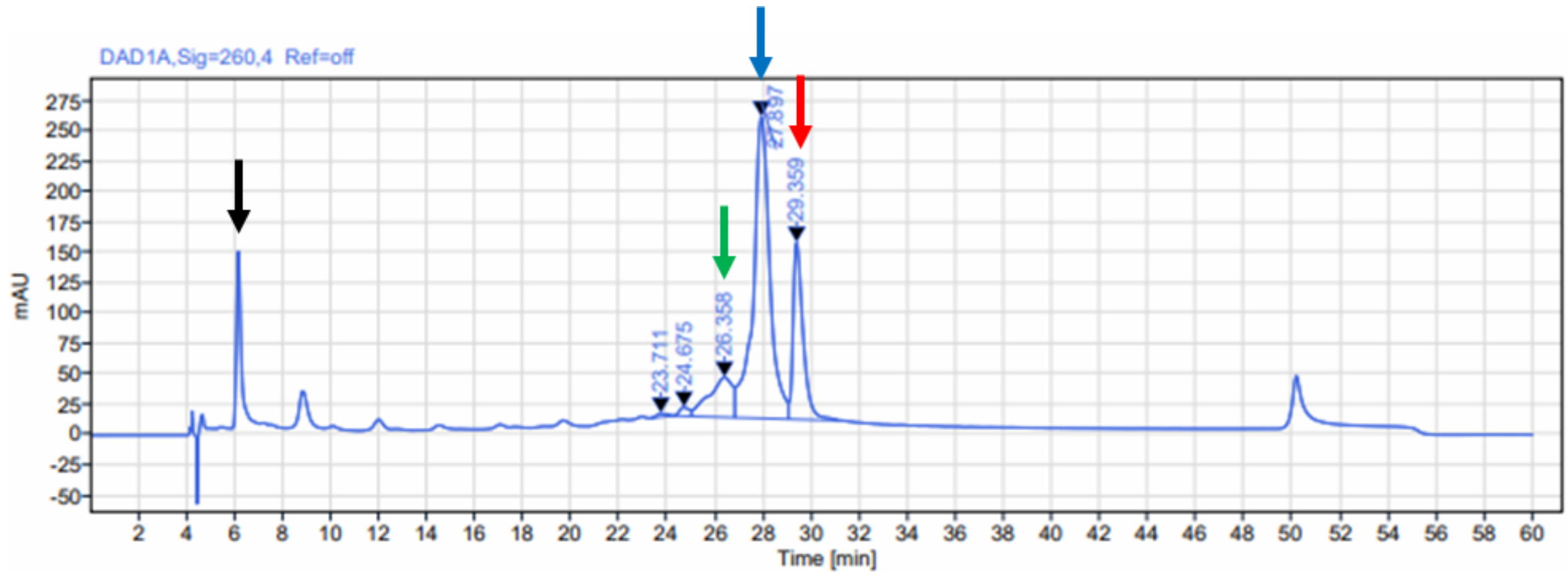

Signal:	DAD1A,Sig=260,4 Ref=off					
**RT [min]**	**Type**	**Width [min]**	**Area**	**Height**	**Area%**	**Name**
23.711	BB	0.94	86.29	2.94	0.46	
24.675	BV	0.76	203.78	7.06	1.09	
26.358	VV	1.80	2210.79	32.57	11.83	
27.897	VV	2.23	11965.05	247.72	64.00	
29.359	VB	2.17	4228.70	145.23	22.62	
		**Sum**	**18694.61**			

B

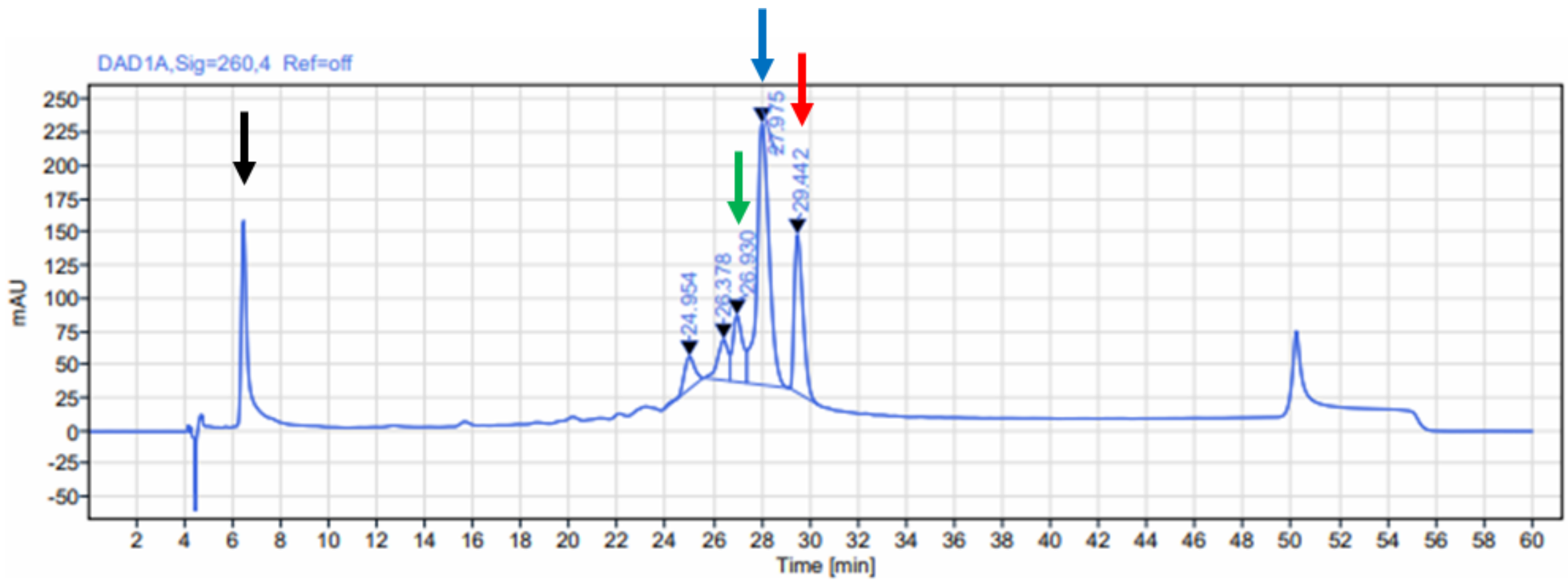

Signal:	DAD1A,Sig=260,4 Ref=off					
**RT [min]**	**Type**	**Width [min]**	**Area**	**Height**	**Area%**	**Name**
24.954	BB	1.67	752.13	24.25	5.79	
26.378	BV	1.11	931.59	30.71	7.17	
26.930	VV	0.69	1451.67	49.98	11.17	
27.975	VB	1.77	6887.24	197.21	53.01	
29.442	BB	1.23	2970.67	119.12	22.86	
		**Sum**	**12993.30**			

Supplementary Figure S9. HPLC-based self-circularization efficiency analysis using peak area integration. Circularization efficiency was calculated as the percentage of circRNA peak area relative to the total peak area within the defined peak region, excluding cleaved ribozyme peaks. STS reaction was performed using the polyA10-IGS P1 construct with the AU11 target site. (**A**) GOI: CVB3-Omicron spike protein (4569-nt). (**B**) GOI: CVB3-dCas9 (4851-nt). Black, green, blue, and red arrows indicate the cleaved group I intron ribozyme, self-cleaved RNA, intact precursor, and circRNA, respectively. The red box indicates the circRNA peak area.

A

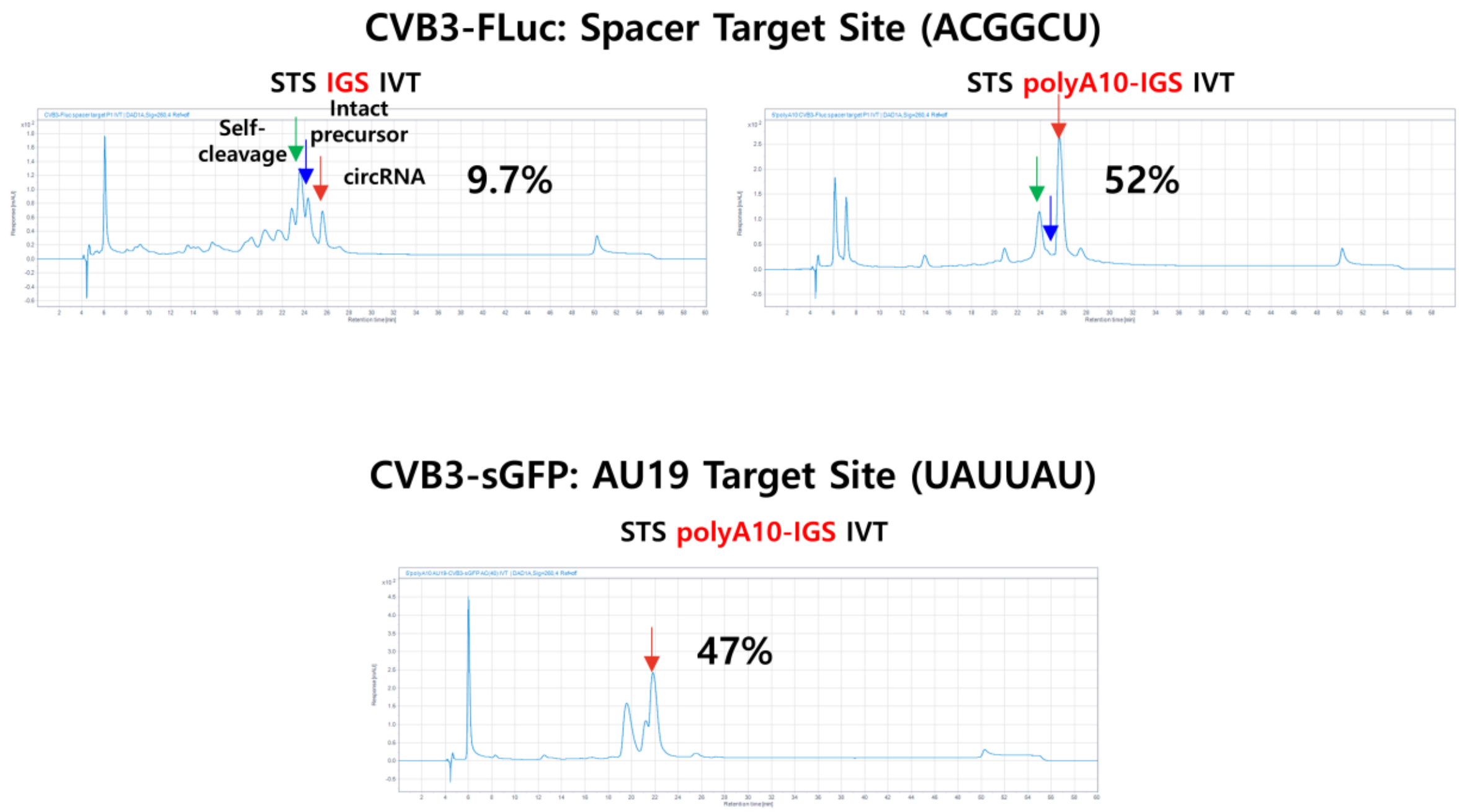

B

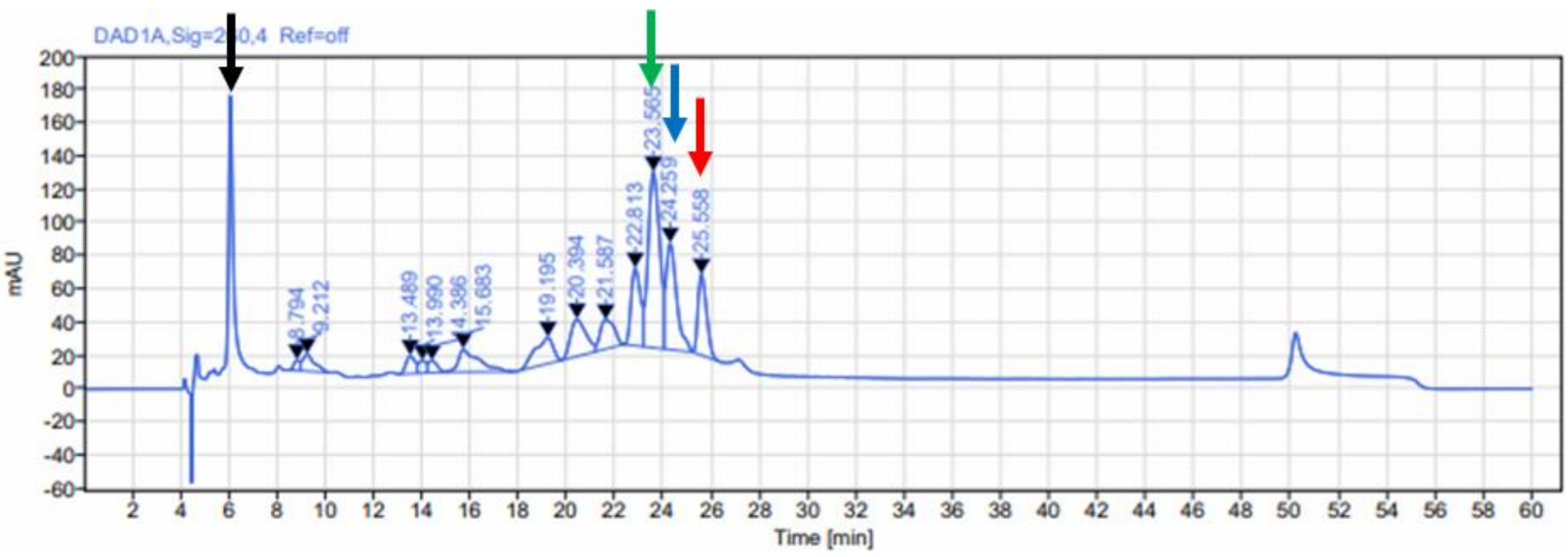

Signal: DAD1A,Sig=260,4 Ref=off

RT [min]	Type	Width [min]	Area	Height	Area%	Name
8.794	MM m	0.47	106.40	6.19	0.86	
9.212	VB	1.23	351.35	10.06	2.84	
13.489	BV	0.75	274.18	10.71	2.21	
13.990	VV	0.42	170.16	7.33	1.37	
14.386	MM m	0.75	180.91	6.95	1.46	
15.683	BB	2.83	807.33	13.62	6.52	
19.195	BB	1.83	836.08	15.59	6.75	
20.394	BV	1.39	982.77	22.21	7.93	
21.587	VB	1.18	715.15	17.25	5.77	
22.813	BV	0.81	1261.93	46.45	10.19	
23.565	VV	0.86	3529.22	105.69	28.49	
24.259	VB	1.18	1968.30	63.84	15.89	
25.558	BB	1.13	1202.80	48.46	9.71	
		Sum	12386.59			

C

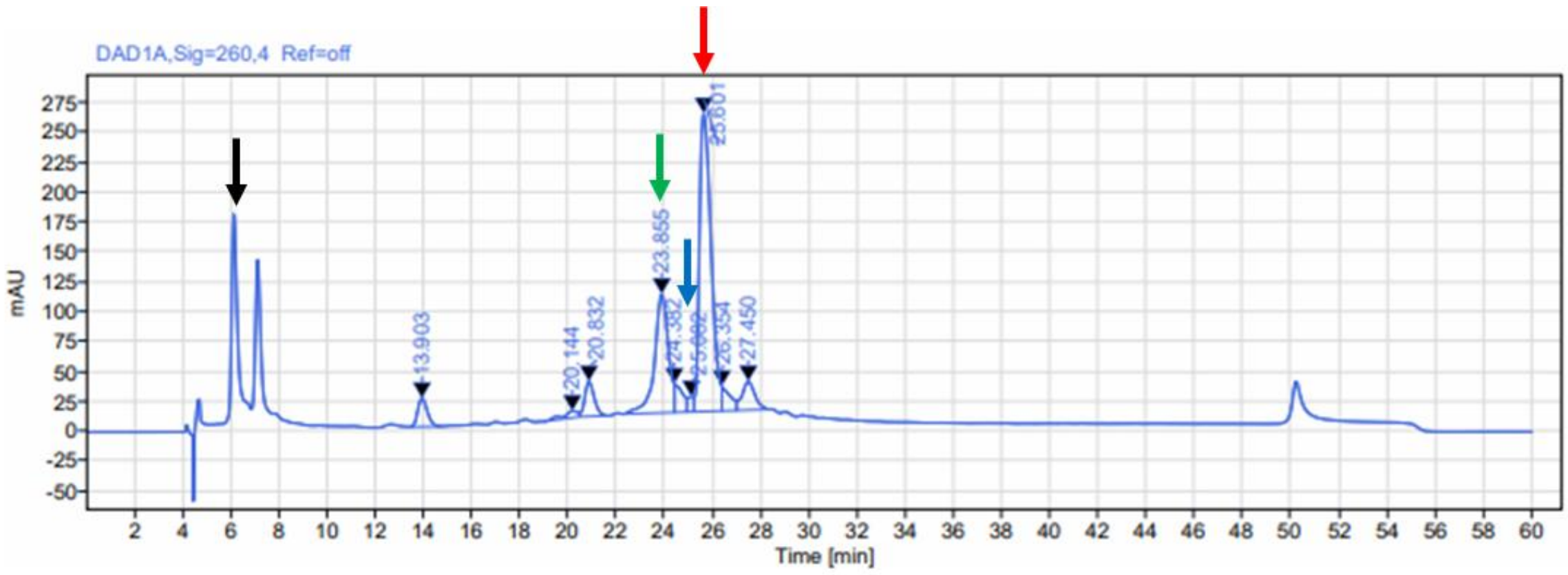

Signal: DAD1A,Sig=260,4 Ref=off

RT [min]	Type	Width [min]	Area	Height	Area%	Name
13.903	BB	1.34	654.70	23.59	4.04	
20.144	MM m	1.47	236.38	5.35	1.46	
20.832	VB	1.17	792.86	29.00	4.89	
23.855	BM m	2.14	3956.05	99.05	24.42	
24.382	MV m	0.52	554.75	23.60	3.42	
25.082	VV	0.29	212.87	12.69	1.31	
25.601	VM m	1.16	8419.68	249.03	51.97	
26.354	MV m	0.61	486.08	20.33	3.00	
27.450	VB	1.37	886.89	23.71	5.47	
		**Sum**	**16200.26**			

D

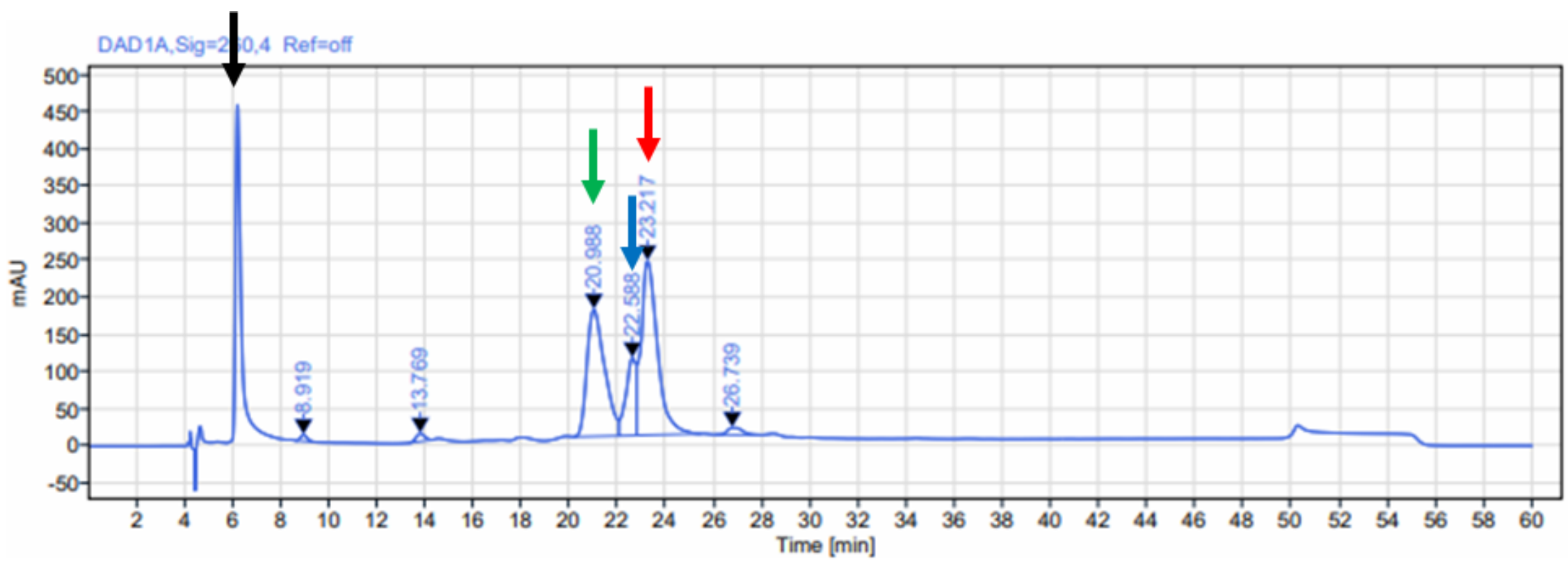

Signal: DAD1A,Sig=260,4 Ref=off

RT [min]	Type	Width [min]	Area	Height	Area%	Name
8.919	BB	0.78	152.50	8.13	0.64	
13.769	BB	0.95	264.42	10.45	1.11	
20.988	BV	1.89	8772.11	170.94	36.92	
22.588	VV	0.74	3046.68	104.19	12.82	
23.217	VB	2.66	11113.37	234.81	46.77	
26.739	BB	1.90	413.67	9.28	1.74	
		**Sum**	**23762.74**			

Supplementary Figure S10. HPLC-based self-circularization efficiency analysis using peak area integration. (**A**) Circularization efficiency was calculated as the percentage of circRNA peak area relative to the total peak area within the defined peak region, excluding cleaved ribozyme peaks. STS reactions were performed using either the P1 construct or the polyA10-IGS P1 construct using spacer target site or AU19 target site. (**B**) GOI: CVB3-FLuc using the P1 construct and spacer target site. (**C**) GOI: CVB3-FLuc using the polyA10-P1 construct and spacer target site. (**D**) GOI: CVB3-sGFP using the polyA10-P1 construct and AU19 target site. Black, green, blue, and red arrows indicate the cleaved group I intron ribozyme, self-cleaved RNA, intact precursor, and circRNA, respectively. The red box indicates the circRNA peak area.

A

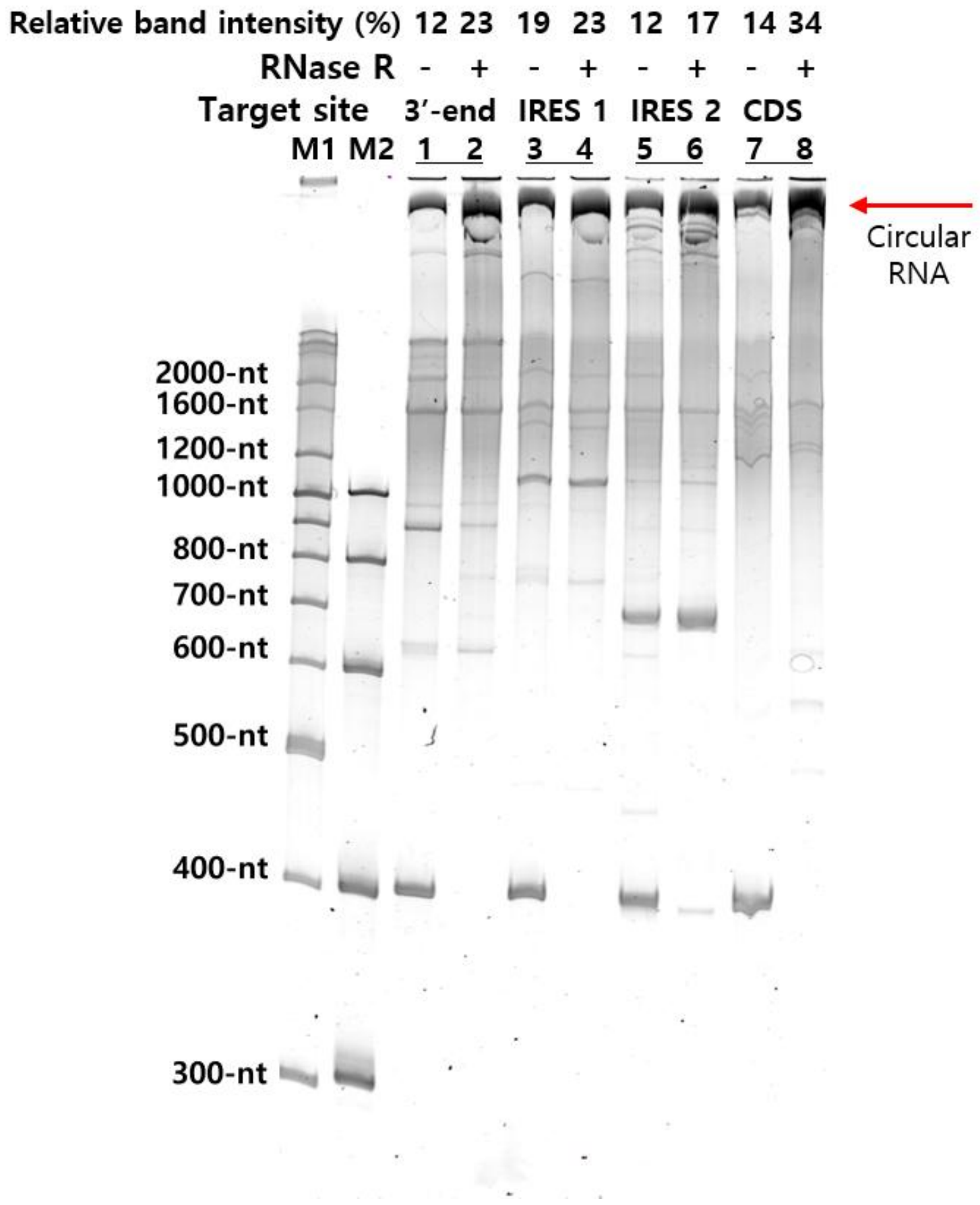

B

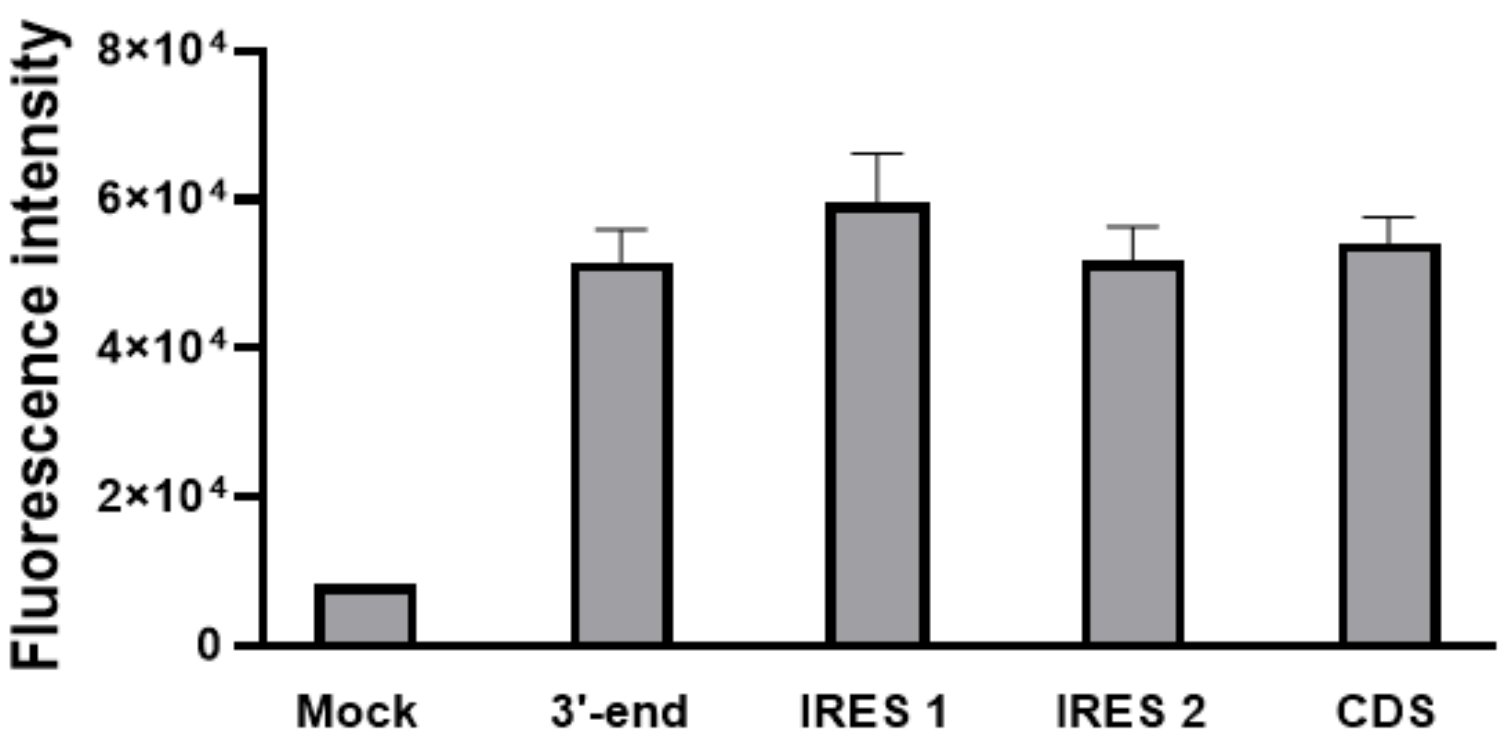

Supplementary Figure S11. Expression analysis of CVB3-sGFP-AC70 circRNAs generated using the different target sites located at various positions within the GOI, as shown in Fig. 2. (**A**) 4% denaturing PAGE analysis of IVT samples generated using the polyA10-IGS strategy before (lane 1, 3, 5 and 7) and after RNase R treatment (lane 2, 4, 6 and 8). (**B**) Reporter protein expression from CVB3-sGFP-AC70 circRNAs generated using different target sites within the GOI in HEK293A cells. Fluorescence intensity was measured 24 hours after transfection.

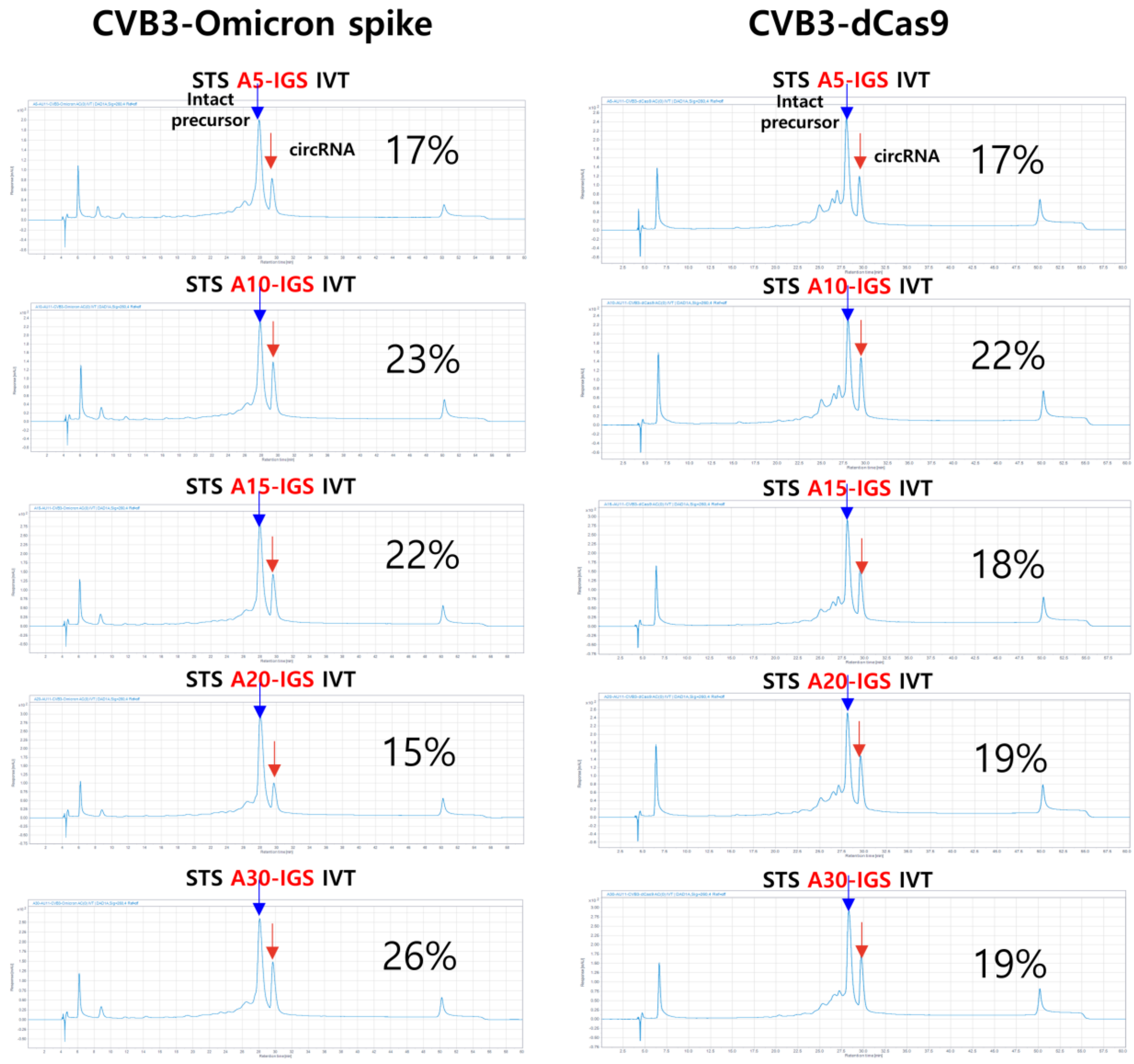

Supplementary Figure S12. HPLC-based self-circularization efficiency analysis using peak area integration. Circularization efficiency was calculated as the percentage of circRNA peak area relative to the total peak area within the defined peak region, excluding cleaved ribozyme peaks. STS reaction was performed using the polyA-IGS P1 constructs containing polyA sequences ranging from 5-nt to 30-nt upstream of IGS, with the AU11 target site. The GOIs were CVB3-Omicron spike protein (4569-nt) and CVB3-dCas9 (4851-nt). Blue and red arrows indicate the intact precursor and circRNA, respectively.

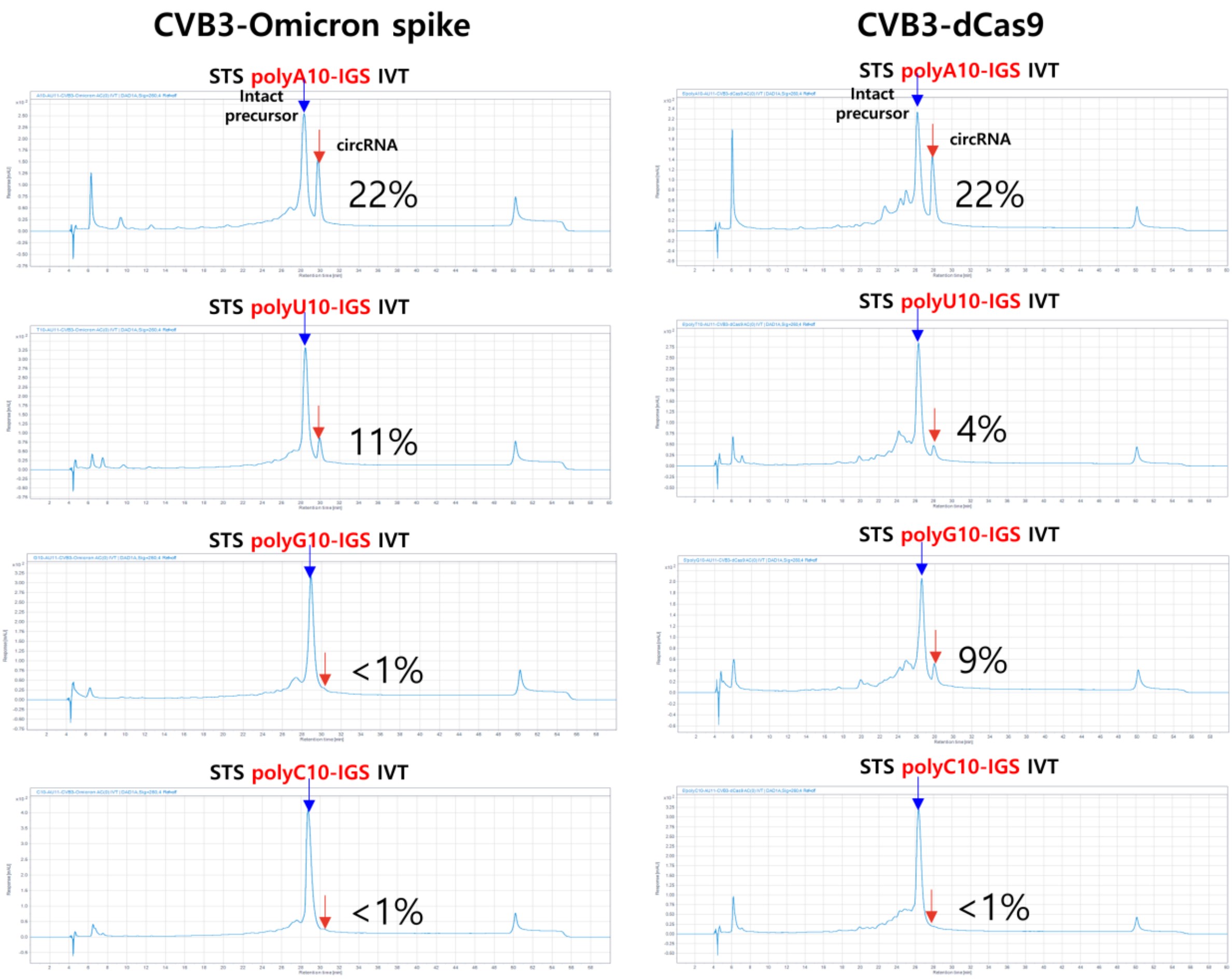

Supplementary Figure S13. HPLC-based self-circularization efficiency analysis using peak area integration. Circularization efficiency was calculated as the percentage of circRNA peak area relative to the total peak area within the defined peak region, excluding cleaved ribozyme peaks. STS reactions were performed using the polyN-IGS P1 constructs containing polyA10, U10, G10, or C10 sequences upstream of IGS, with the AU11 target site. The GOIs were CVB3-Omicron spike protein (4569-nt) and CVB3-dCas9 (4851-nt). Blue and red arrows indicate the intact precursor and circRNA, respectively.

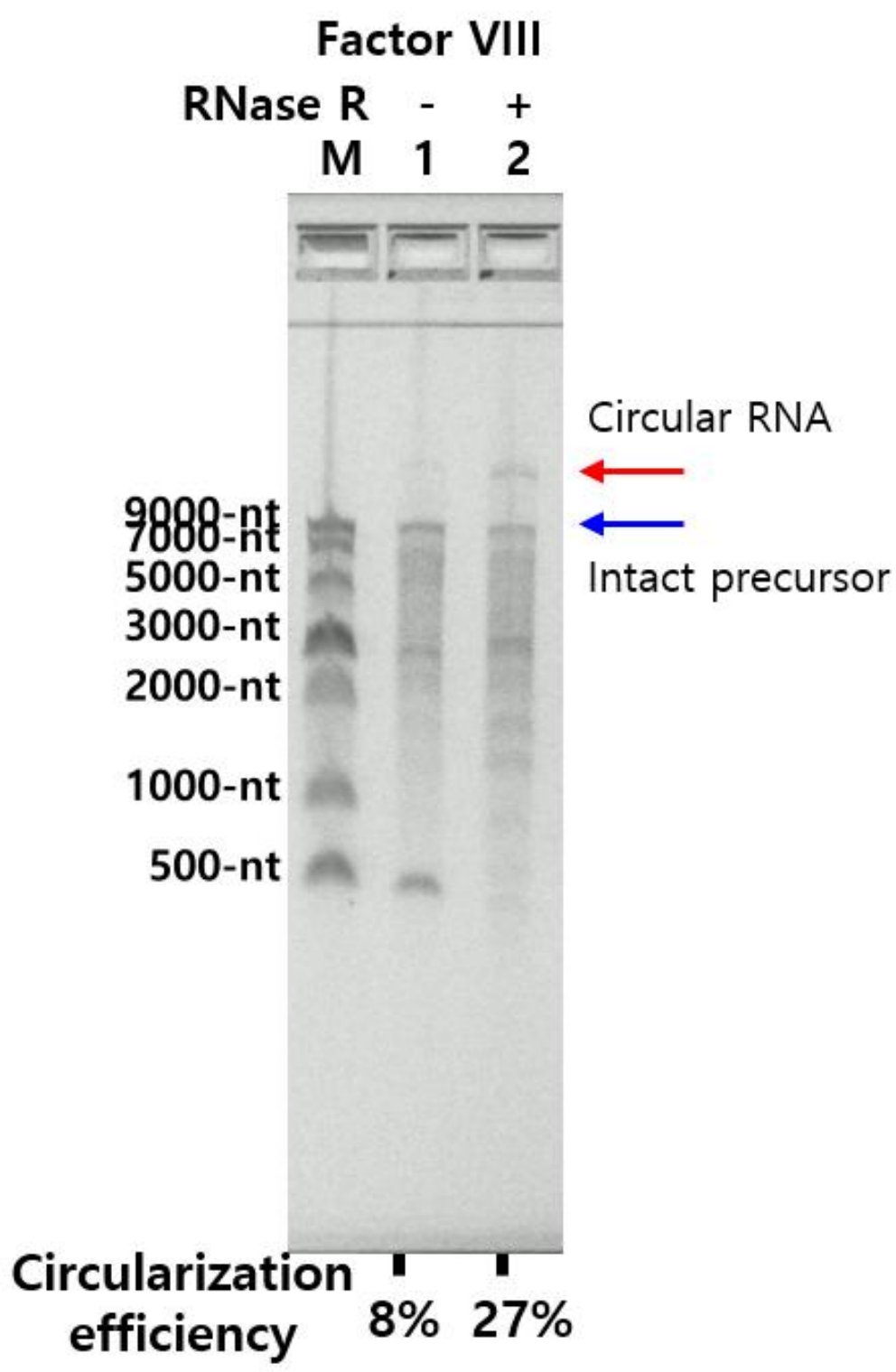

Supplementary Figure S14. 1% E-gel EX agarose gel analysis of IVT samples or RNase R-treated IVT samples from original P1 constructs generating CVB3-Factor VIII circRNA. CVB3-Factor VIII IVT sample (lane 1) or RNase R-treated IVT sample (lane 2). Circularization efficiency was determined based on relative circRNA band intensity.

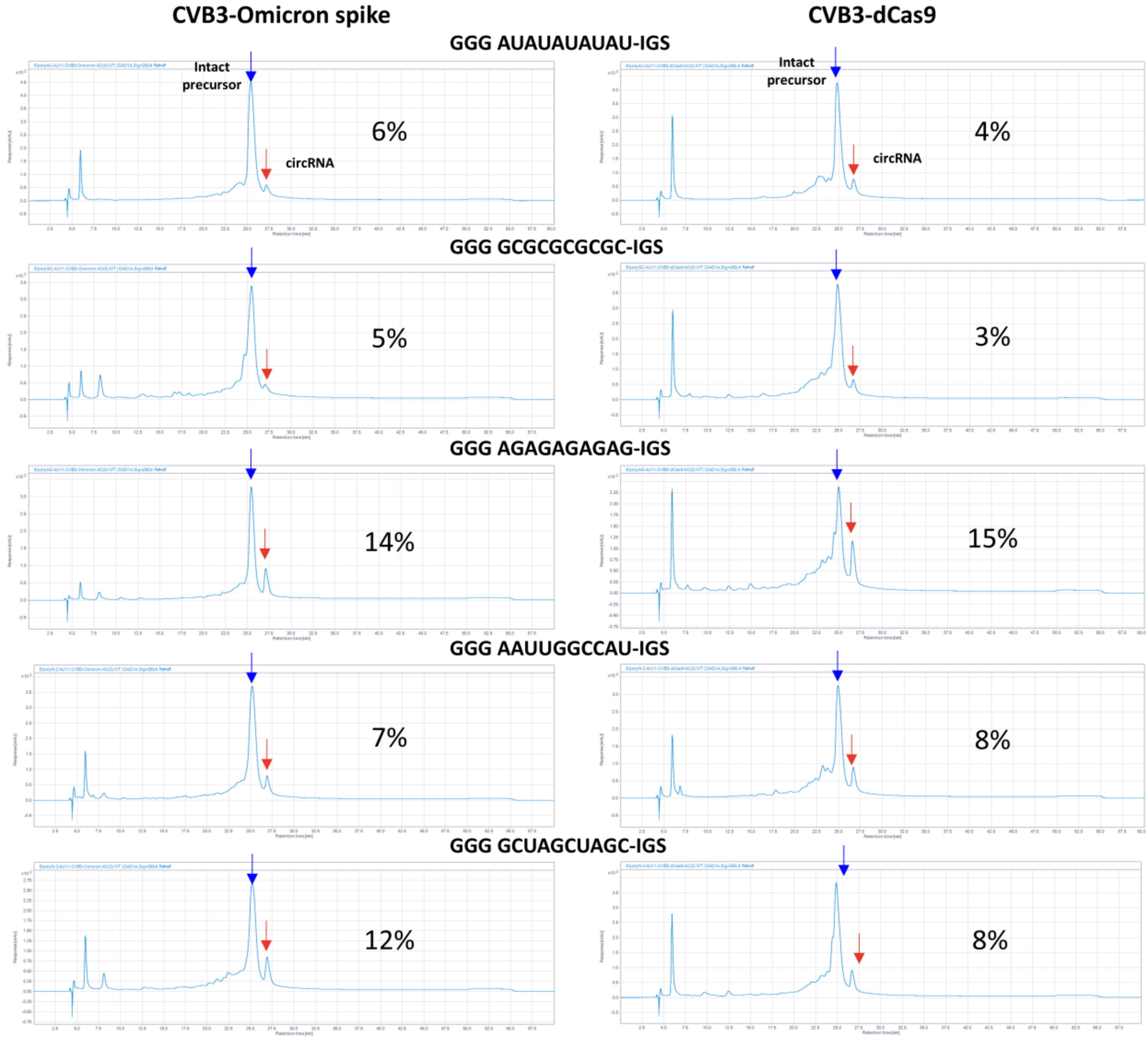

Supplementary Figure S15. HPLC-based self-circularization efficiency analysis using peak area integration. Circularization efficiency was calculated as the percentage of circRNA peak area relative to the total peak area within the defined peak region, excluding cleaved ribozyme peaks. STS reactions were performed using the polyN-IGS P1 constructs containing various 10-nt polyN sequences upstream of IGS, with the AU11 target site, to evaluate sequence-dependent effects on self-circularization. The GOIs were CVB3-Omicron spike protein (4569-nt) and CVB3-dCas9 (4851-nt). Blue and red arrows indicate the intact precursor and circRNA, respectively.

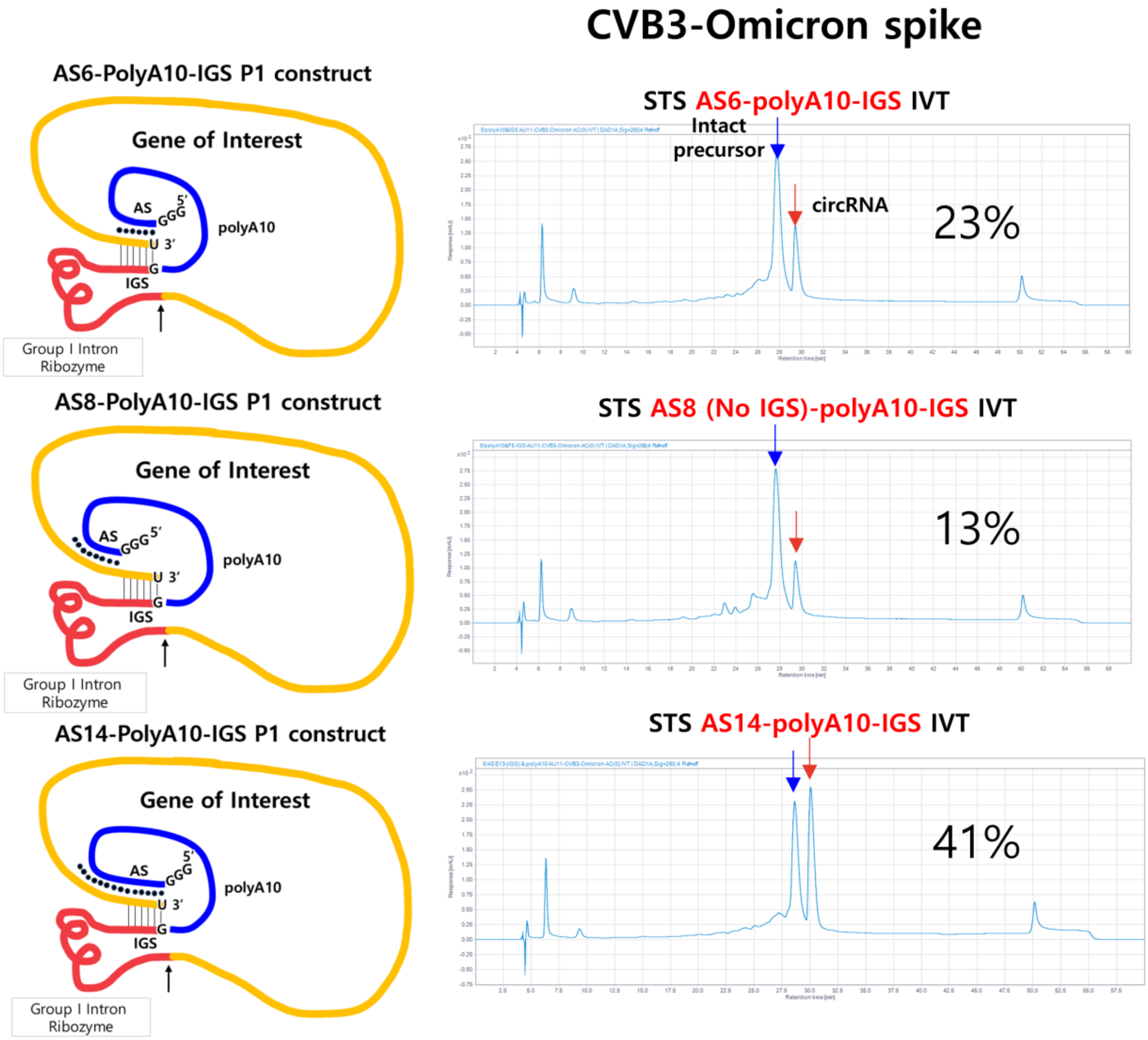

Supplementary Figure S16. HPLC-based self-circularization efficiency analysis using peak area integration. Circularization efficiency was calculated as the percentage of circRNA peak area relative to the total peak area within the defined peak region, excluding cleaved ribozyme peaks. STS reactions were performed using the AS-polyA10-IGS P1 constructs containing AS6, AS8 (No IGS), or AS14 upstream of polyA10, with the AU11 target site. The GOI was the CVB3-Omicron spike protein (4569-nt). Blue and red arrows indicate the intact precursor and circRNA, respectively. Dots indicate complementary base pairing of the AS with the target site and/or its surrounding sequences.

A

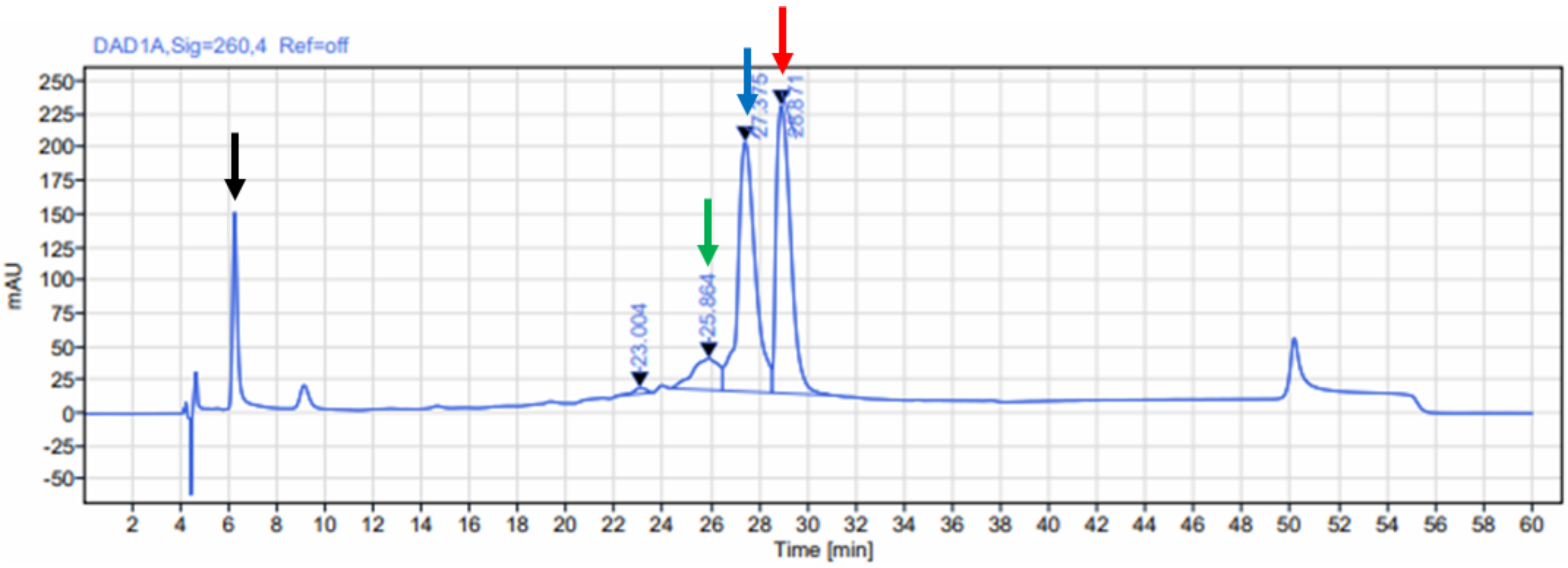

Signal:	DAD1A,Sig=260,4 Ref=off					
**RT [min]**	**Type**	**Width [min]**	**Area**	**Height**	**Area%**	**Name**
23.004	BB	1.50	169.61	4.41	0.83	
25.864	BV	2.15	1797.17	23.57	8.80	
27.375	VV	2.05	9423.95	187.70	46.13	
28.871	VB	2.54	9039.06	216.10	44.24	
		**Sum**	**20429.79**			

B

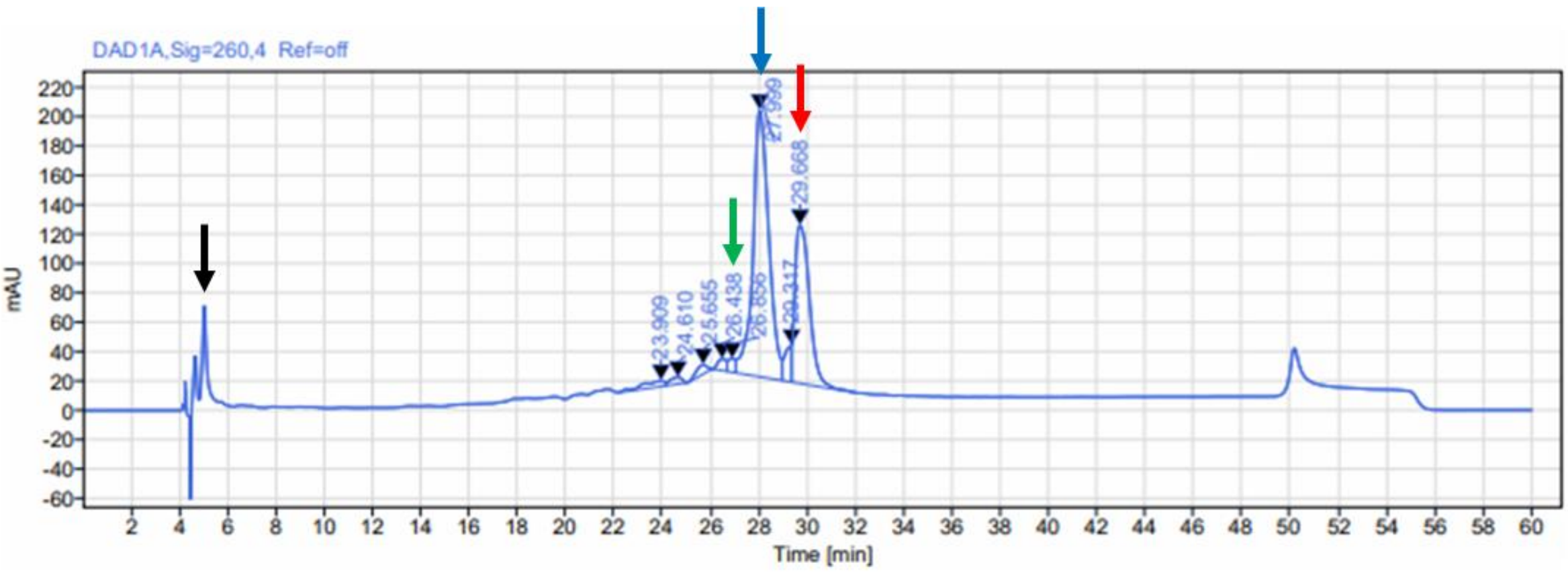

Signal:	DAD1A,Sig=260,4 Ref=off					
**RT [min]**	**Type**	**Width [min]**	**Area**	**Height**	**Area%**	**Name**
23.909	BV	1.95	274.73	3.94	1.91	
24.610	VB	0.87	162.93	4.50	1.13	
25.655	BB	0.99	207.42	6.32	1.44	
26.438	BV	0.61	191.16	7.81	1.33	
26.856	VV	0.36	187.97	9.14	1.31	
27.999	VV	1.92	8335.05	182.06	57.98	
29.317	VM m	0.39	474.84	24.98	3.30	
29.668	MB m	2.66	4541.72	107.87	31.59	
		**Sum**	**14375.81**			

Supplementary Figure S17. HPLC-based self-circularization efficiency analysis using peak area integration. Circularization efficiency was calculated as the percentage of circRNA peak area relative to the total peak area within the defined peak region, excluding cleaved ribozyme peaks. The GOI was the CVB3-Omicron spike protein (4569-nt). (**A**) STS method using the AS-polyA10-IGS P1 construct with the AU11 target site. (**B**) PIE method. Black, green, blue, and red arrows indicate the cleaved group I intron ribozyme, self-cleaved RNA, intact precursor, and circRNA, respectively. The red box indicates the circRNA peak area.

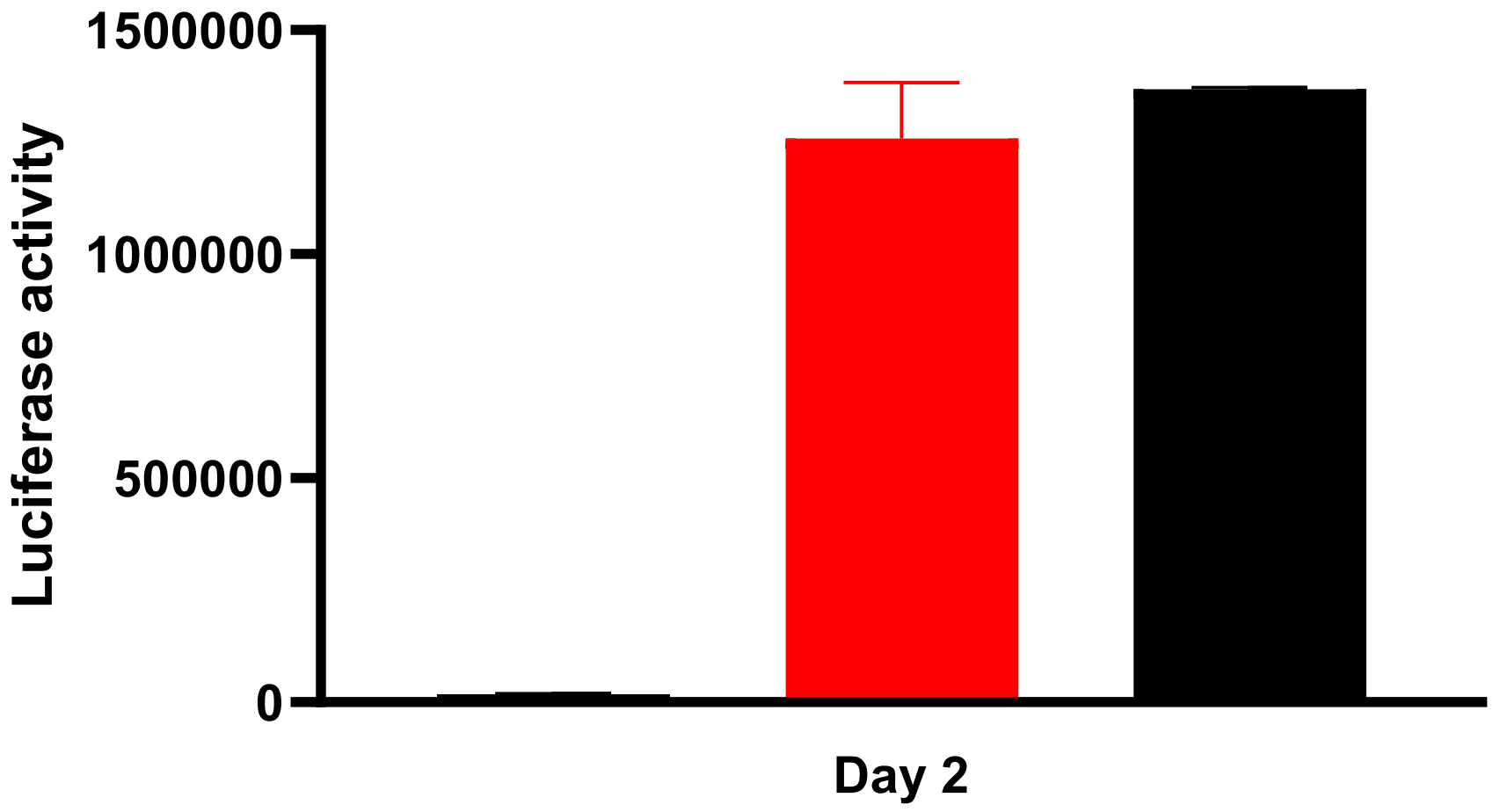

Supplementary Figure S18. Reporter protein expression from HPLC-purified CVB3-FLuc circRNAs generated using the conventional P1 or AS-polyA10-IGS P1 construct with the AU11 target site within the CVB3 IRES (GOI: CVB3 IRES-FLuc-AC40) in HEK293A cells. Firefly luciferase activity was measured at 2 days post-transfection. White bars, mock; red bars, conventional P1; black bars, AS-polyA10-IGS P1.

A

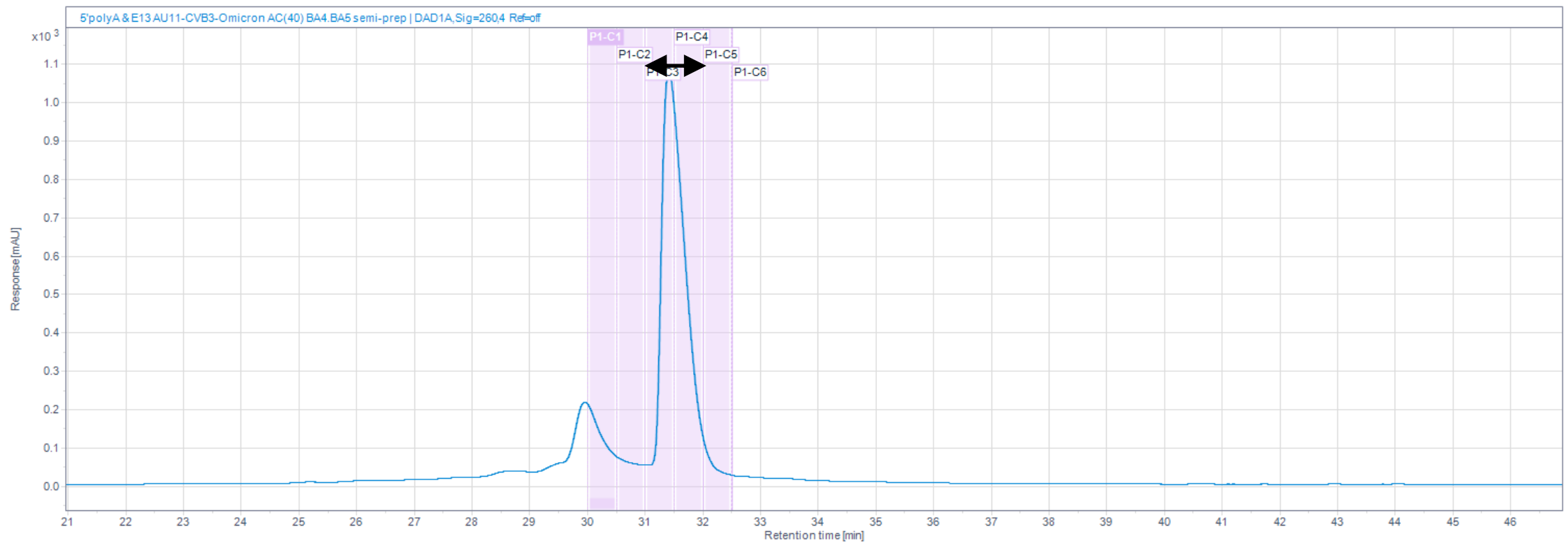

B

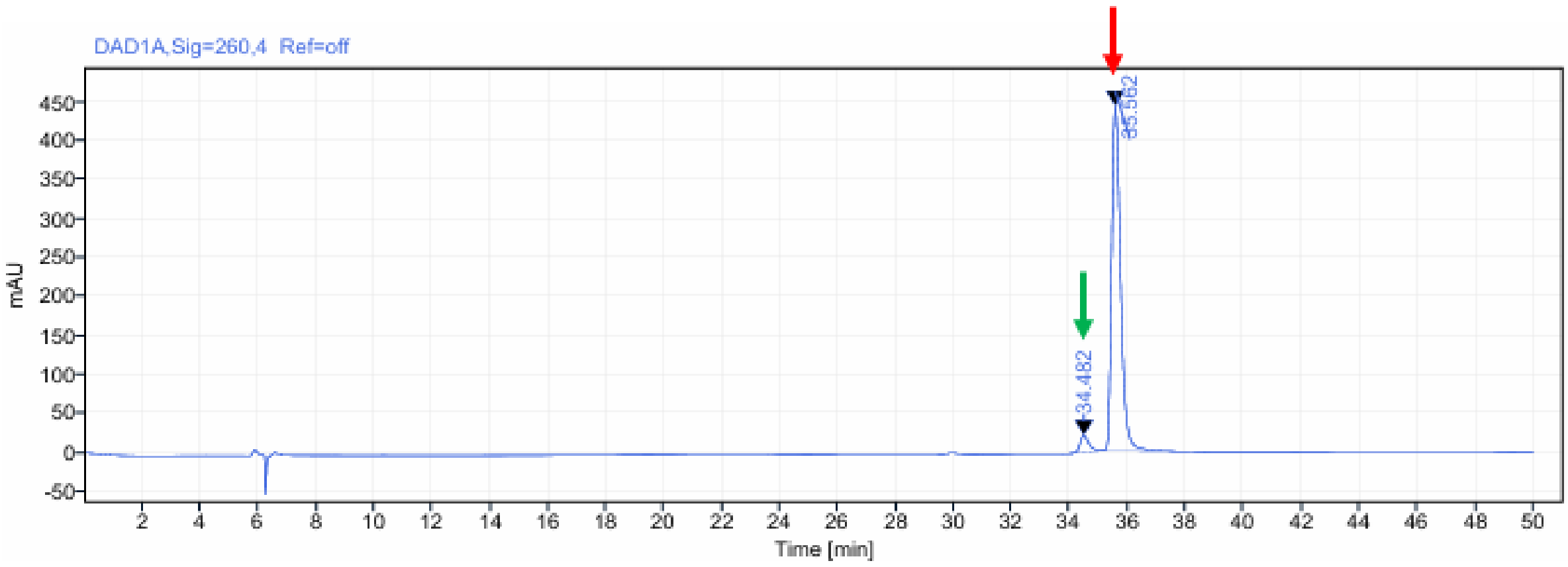

Signal: DAD1A,Sig=260,4 Ref=off

RT [min]	Type	Width [min]	Area	Height	Area%	Name
34.482	BB	1.06	452.42	21.26	4.56	
35.562	BB	2.96	9462.94	441.00	95.44	
		**Sum**	**9915.36**			

Supplementary Figure S19. IP-RP HPLC purification of RNase R-treated circRNA. The GOI was the CVB3-Omicron spike protein (4569-nt). (**A**) Representative IP-RP HPLC chromatogram during purification. The arrow indicates the circRNA peak collected for purification. The collected fraction was recovered by standard ethanol precipitation method. (**B**) Representative IP-RP chromatogram of the purified circRNA, showing >95% purity. Green and red arrows indicate nicked circRNA and intact circRNA, respectively. The red box indicates the circRNA peak area.
